# Hypoxia represses microRNA biogenesis proteins in breast cancer cells

**DOI:** 10.1186/1471-2407-14-533

**Published:** 2014-07-22

**Authors:** Veronika Bandara, Michael Z Michael, Jonathan M Gleadle

**Affiliations:** 1 Renal Department, Flinders Medical Centre, Flinders University School of Medicine, Bedford Park, Adelaide, South Australia 5042, Australia; 2 Department of Gastroenterology and Hepatology, Flinders Medical Centre, Flinders University School of Medicine, Bedford Park, Adelaide, South Australia 5042, Australia

**Keywords:** Hypoxia, MicroRNA, Breast cancer, Dicer, Drosha, Oxygen

## Abstract

**Background:**

Cancers are commonly characterised by hypoxia and also by global reductions in the levels of mature microRNAs. We have examined the hypothesis that hypoxia might mediate this reduction through repressive effects on microRNA biogenesis proteins.

**Methods:**

Breast cancer cell lines were exposed to hypoxia and manipulations of hypoxia inducible factor (HIF) and HIF hydroxylase activity. The effects of hypoxia on the mRNA and protein levels of enzymes involved in microRNA biogenesis (Dicer, Drosha, TARPB2, DCGR8, XPO5) was determined by RT PCR and immunoblotting. The effect of hypoxia on microRNAs was determined with microarray studies, RT PCR and reporter assays.

**Results:**

In breast cancer lines there was significant reduction of Dicer mRNA and protein levels in cells exposed to hypoxia. This effect was independent of HIF but dependent on the HIF hydroxylase PHD2 and was partly mediated by feedback effects via microRNAs. Furthermore, several other proteins with critical roles in microRNA biogenesis (Drosha, TARBP2 and DCGR8) also showed significant and co-ordinated repression under hypoxic conditions. Despite these substantial alterations no, or modest, changes were observed in mature microRNA production.

**Conclusion:**

These observations provide further and important interfaces between oxygen availability and gene expression and a potential mechanistic explanation for the reduced levels of microRNAs observed in some cancers. They provide further support for the existence of feedback mechanisms in the regulation of the microRNA biogenesis pathway and the relative stability of microRNAs.

## Background

Hypoxia is a key feature of many cancers and the presence of hypoxia is associated with more aggressive and metastatic tumours [1-3]. The exposure of cells to hypoxia leads to the co-ordinated regulation of many genes. The protein products of these genes have a wide variety of critical roles in processes such as metabolism, angiogenesis, growth and apoptosis. Studies of the mechanisms underlying the regulation of such genes have implicated a central role for the transcription factor hypoxia inducible factor (HIF). Many cancers are characterised by enhanced HIF levels and increased expression of hypoxically regulated genes which correlate both with tumour aggression and patient outcome [4]. The extent to which hypoxia contributes to enhanced tumour aggression via metabolic alterations, changes in gene expression and/or epigenetic modifications remains incompletely understood. In addition to the transcriptional regulation of mRNA, hypoxia can also influence mRNA stability, translation, protein stability and microRNA generation. Hypoxia also leads to the co-ordinated repression of many genes but the mechanism for such effects is less well defined.

MicroRNAs (miRNAs) are 17–22 nucleotide, non-coding, single stranded RNA molecules that are important regulators of gene expression. MicroRNAs are transcribed as primary transcripts (pri-miRNAs) by RNA Polymerase II [5] or III [6] enzymes. In the nucleus, pri-miRNAs are cleaved by the nuclear RNase III enzyme Drosha and cofactor protein DGCR8 (DiGeorge syndrome critical region gene 8) to generate a precursor miRNA (pre-miRNA) (about 70 nucleotides long) [7]. The pre-miRNAs are exported to the cytoplasm by the karyopherin, exportin-5 [8]. In the cytoplasm, pre-miRNAs are further cleaved by Dicer, a ribonuclease III enzyme [9,10], coupled with TARBP2 (Tar RNA binding protein 2) [11,12] to generate a 22 nucleotide double stranded miRNA duplex. One strand of the RNA duplex functions as the mature miRNA and often the passenger strand will be degraded by endonucleases [13]. In some instances the passenger strand (previously referred to as the star form) is not degraded and may also function as a mature miRNA [14,15]. The two strands of the duplex are separated by an RNA helicase (DDX5) and the miRNA then binds to mRNA within the RNA induced silencing complex, containing an Argonaute protein, leading to either translational inhibition or destruction of the target mRNA [16].

Functional studies show that miRNAs are involved in regulating many cellular processes including developmental timing, cell differentiation, cell proliferation and cell death [17,18]. The expression levels of many miRNAs are deregulated in human disease conditions including cancer [19-23]. In addition to deregulation of specific miRNAs in cancer, it has emerged that most tumour cell lines and cancers are characterised by global reductions in miRNA expression [24,25] when compared to adjacent normal tissue. Whilst it has been postulated that this reduction in miRNA expression is a feature of a loss of differentiation, the mechanisms underlying this global reduction in miRNA expression in many cancers are unknown, though some evidence exists for post-transcriptional control [25,26].

It has recently been suggested that genetic polymorphisms of the genes encoding miRNA generating proteins may affect renal cell carcinoma susceptibility [27]. Indeed for other cancers such as ovarian and lung tumours, low levels of Drosha [28] and Dicer [28,29] have been shown to correlate with poor patient outcome. Dicer1 is a haploinsufficient tumour suppressor in human cancer and loss of one Dicer1 allele is sufficient for the formation of tumours in breast, kidney, stomach, intestine, liver, lungs and pancreas [30]. Furthermore, miRNAs are central to the process of angiogenesis (for review see [31]). Exposure to hypoxia alters specific miRNA expression [32-35] with miRNAs such as miR-210 showing marked hypoxic induction and capacity to act as markers of patient prognosis in breast cancer [35].

The links between tumour hypoxia, miRNA expression and cancer aggression raise the possibility of a general effect of hypoxia on miRNA biogenesis and function. During our microarray study examining mRNA expression in the breast cancer cell line MCF7, we saw a modest but consistent decrease in Dicer mRNA levels after exposure to hypoxia [36]. In this work we investigate the possibility that hypoxic regulation of expression of miRNA biogenesis proteins might contribute to the reduction in miRNA expression in many tumours and to the role of hypoxia in cancer progression.

## Methods

### Cell culture

Breast cancer cell lines (MCF7 and SKBR3) and colorectal cancer cell line (HT29) were obtained from the American Type Culture Collection (Manassas, VA, USA). The collection of primary human umbilical vein endothelial cells (HUVEC) for use in this study was given ethical clearance from the Royal Adelaide Hospital (RAH), Adelaide, South Australia. Consent was obtained from all subjects in accordance with the ‘Declaration of Helsinki’ and conforms to the guidelines established by the National Health and Medical Research Council of Australia.

Breast cancer cell lines (MCF7 and SKBR3) and colorectal cancer cell line (HT29) were maintained in RPMI 1640 (Invitrogen) medium supplemented with 10% foetal bovine serum (FBS) (Bovigen). Primary cell line human umbilical vein endothelial cells (HUVECs) were maintained in polystyrene flasks coated with gelatine in Media 200 PRF (Invitrogen) supplemented with 20% FBS (Bovigen). Cells were maintained at 37°C with 5% CO_2_. All experiments were conducted in triplicate with independent cell cultures. Independently treated cultures were treated as technical replicates.

### Cell treatments

Cells were treated with 1 mM dimethyloxalylglycine (DMOG) (Enzo Life Sciences) and incubated at 37°C in a normoxic incubator for 48 h. Similarly cells were treated with 0.1 mM Desferrioxamine (Sigma-Aldrich) and incubated at 37°C in a normoxic incubator for 48 h.

### Exposure of cells to hypoxia

A hypoxic incubator (Coy Laboratory Hypoxic workstation glove box) was used to expose cells to continuous controlled hypoxic conditions. This humidified, temperature controlled (37°C) chamber was supplemented with 5% CO_2_, and N_2_ (as required to maintain controlled O_2_ levels). Cells were exposed to 0.1% O_2_ levels or 1% O_2_ for varying durations. Normoxic controls were incubated in parallel in a humidified incubator supplemented with 5% CO_2_ at 37°C.

### RNA interference

Cells were seeded at 5 × 10^4^ cells per well in 24-well plates (1.9 cm^2^) and grown for 24 h. Cells were transfected with 20 nM siRNA duplexes (Shanghai GenePharma Co., Ltd, China), using Lipofectamine 2000 reagent (Invitrogen) following the manufacturer’s protocol. A second transfection was carried out after 24 h following the same protocol. Cells were harvested 24 h after the second transfection and used for RNA and protein extraction. List of siRNAs used are provided (see Additional file 1: Table S1).

### Transfection with miRNA mimics and inhibitors

Cells were seeded at 5 × 10^4^ cells per well in 24-well plates and grown for 24 h. Cells were transfected with 20 nM miRNA mimic, inhibitors or antagomirs (Shanghai GenePharma Co., Ltd, China), using Lipofectamine 2000 reagent (Invitrogen) following the manufacturer’s protocol. Cells were harvested 24 h after the transfection and used for RNA and protein extraction.

### Plasmid DNA transfection

A plasmid construct containing the 3’UTR of the ZEB1 gene, downstream of *Renilla luciferase* (RL), in a CMV promoted RL reporter (pCI-neo-hRL) was used [37]. ZEB1 is an E-cadherin transcriptional repressor involved in epithelial to mesenchymal transition of tissues and is regulated by the mir-200 family. To over-express miR-200b levels we used a plasmid expressing a precursor miR-200b from a CMV promoter (pCMV-miR-200b) and an empty vector was used as the control plasmid (Origene).

The RL reporter plasmids (3.6 fmol), pGL3-control (Promega) (500 ng for normalisation) and pCMV-miR-200b/pCMV-miR plasmid (250 ng) were co-transfected with Lipofectamine 2000 (Invitrogen) into SKBR3 cells seeded in 24-well plates (1 × 10^5^ cells per well). The total amount of DNA in each transfection was made up to 1 μg with unrelated plasmid DNA (pcDNA 3.1+). Two hours after transfection, half the plates were incubated at 0.1% O_2_ for 24 h and control plates were incubated at normoxic conditions. After the 48 h incubation cells were assayed using the dual-luciferase reporter assay system (Promega). All experiments were performed in triplicate. Luminescence was measured using a plate reader luminometer (Beckman Coulter DTX 880 Multimode detector).

### RNA extraction and real time PCR

Total RNA was extracted using TRIzol reagent (Invitrogen) following the manufacturers protocol. RNA quantity and quality were determined using a Nanodrop-8000 spectrophotometer (Nanodrop Technology) and Agilent 2100 Bioanalyzer. For miRNA analysis complementary DNA (cDNA) was synthesised from 5 ng of total RNA using Taqman miRNA specific primers and Taqman miRNA reverse transcription kit (Applied Biosystems). Small nucleolar RNA, RNU6B, was used as a control gene. For parallel detection of mature and pre-miRNAs, cDNA was synthesised from 1 μg of RNA using the miScript II RT kit. Mature miRNAs were analysed using miScript primer assays and precursor miRNAs were analysed using miScript precursor assays. For mRNA analysis cDNA was randomly primed from 1 μg of total RNA following DNase 1 treatment (New England Labs) using M-MLV reverse transcriptase RNase H minus, point mutant (Promega) and Random primer 6 (New England Labs). Real time PCR was subsequently performed in triplicate with 1:5 dilution of cDNA using Taqman gene expression assays. Beta-2-microglobulin and ribosomal RNA 18S were used to normalise mRNA expression. Relative quantification by RT-PCR was performed using the Corbett Roto-gene 6000 and data analysed using Corbett Rotogene software (Version 5.0.61) (Corbett Research).

### Microarray analysis

Total RNA from MCF7 cells, exposed to hypoxia or normoxia, was extracted using the TRIzol protocol as above. RNA integrity was assessed using the Agilent 2100 Bioanalyzer. Affymetrix miRNA 3.1 Array Strip was used for RNA analysis. This array consisted probe sets unique to human mature and pre-miRNA hairpins. A detailed protocol can be found in the miRNA 3.1 Array Strips technical manual (Affymetrix). In summary, 100–300 ng of total RNA was used to synthesise double stranded cDNA using random hexamers. The cDNA was then amplified to produce antisense cRNA, which was then reverse transcribed in a second cycle of cDNA synthesis. The second cycle incorporates dUTP into the cDNA sequence, which allows it to be fragmented using uracil DNA glycosylase and apurinic/apyrimidic endonuclease I. Following biotinylation, these fragments were hybridised overnight to a Affymetrix miRNA 3.1 array. The arrays were then washed, stained using a fluorescently-labelled antibody, and scanned using a high-resolution scanner. Intensity data were analysed using Partek® software (Partek Inc.). Data were normalised by quantile normalisation and log_2_ transformed. Differential expression was determined by ANOVA and corrected for false discovery. The microarray data have been deposited in NCBI’s Gene Expression Omnibus [38] and are accessible through GEO Series accession number GSE49999.

### Immunoblotting

Cell lysates were prepared by adding lysis buffer (6.7 M urea, 10 mM Tris–HCl (pH 6.8), 10% glycerol, 1% SDS, supplemented with 1 mM DTT and Complete mini protease inhibitor cocktail tablets (Roche Applied Science, UK) just before use, to cells washed with 1× phosphate buffered saline solution. Total protein extracts were resolved on an 8% SDS polyacrylamide gel or a Mini-PROTEAN TGX precast Any kD gradient gel (Bio-Rad Laboratories, Inc.) electrophoresis and transferred to a polyvinylidene difluoride membrane (Millipore). The use of Mini-PROTEAN TGX precast gels allowed for imaging the total protein loaded in each lane on the gel and on the PVDF membrane after the transfer. The membrane was blocked with 5% skim milk in 1xTBS-T for 1 h at room temperature and incubated with primary antibody overnight at 4°C, then washed with 1xTBS-T and incubated with the appropriate horseradish peroxidise conjugated secondary antibodies for 1 h at room temperature. The enhanced chemiluminescence (ECL) (GE health care) system was used to detect bands, which were imaged with a Chemi-Doc MP system (Bio-Rad). Densitometry was performed using the ImageLab software version 4.0 (BioRad). Protein levels were normalised to β-tubulin, α-actinin or to total protein levels on the PVDF membrane after the transfer. Quantified results represent n = 3 samples. Statistical significance determined using Student’s t-test (P < 0.05).

## Results

### The effect of hypoxia on Dicer protein levels in breast cancer cell lines

**Figure 1:**
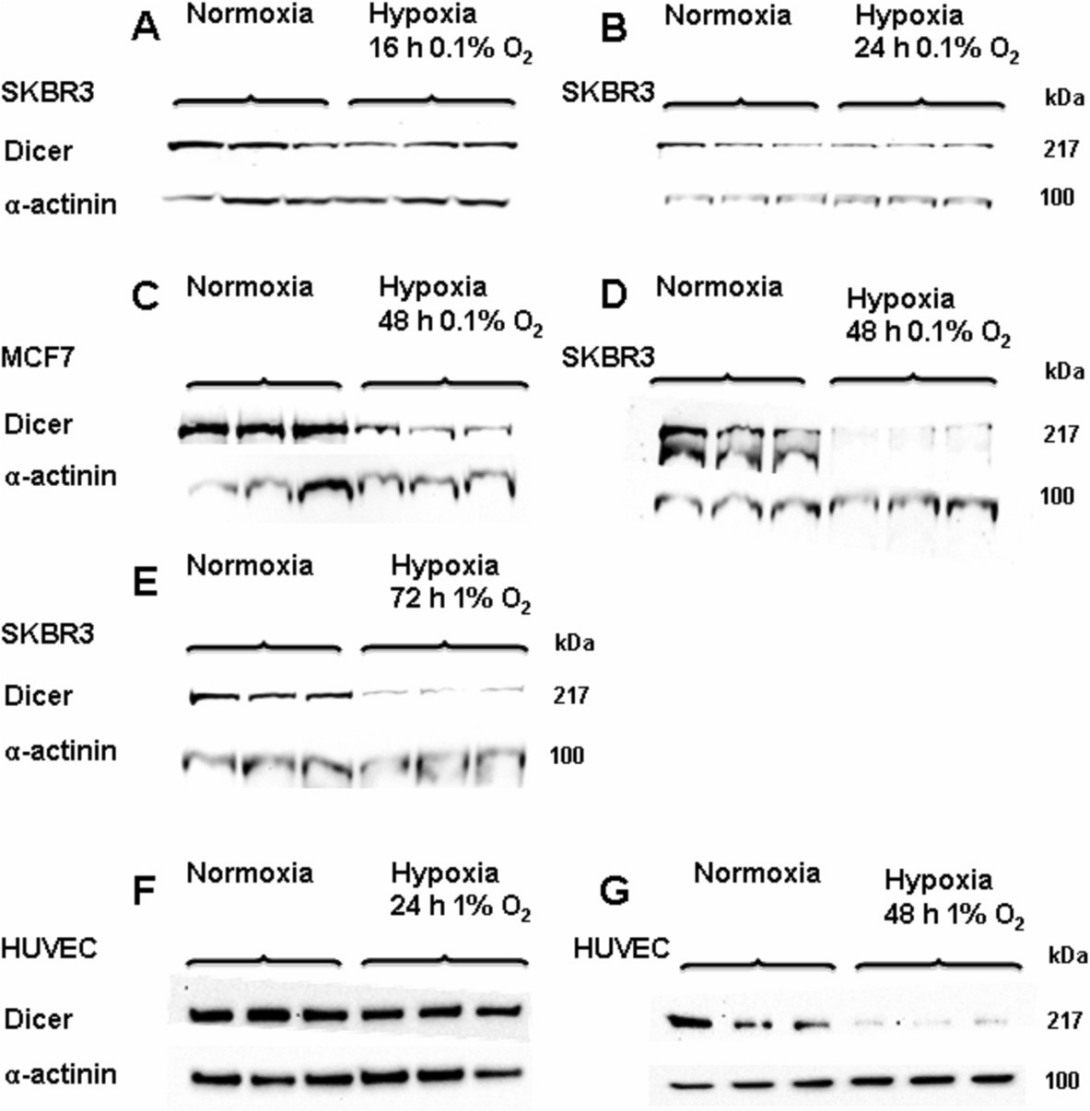
**Dicer protein expression in hypoxia vs. normoxia.** Dicer protein expression was examined in SKBR3, MCF7 and HUVEC cells after exposure to different O_2_ concentrations for different durations. **A**, Dicer protein expression in SKBR3 cells after exposure to 0.1% O_2_ for 16 h and **B**, 24 h. **C**, Dicer protein expression in MCF7 cells after exposure to 0.1% O_2_ for 48 h. **D**, Dicer protein expression in SKBR3 cells after exposure to 0.1% O_2_ for 48 h. **E**, Dicer protein expression in SKBR3 cells after exposure to 1% O_2_ for 72 h. **F**, Dicer protein expression in HUVECs after exposure to 1% O_2_ for 24 h. **G**, Dicer protein expression in HUVECs after exposure to 1% O_2_ for 48 h (P = 0.1). Dicer and α-actinin protein expression was examined by immunoblotting. Results show three technical replicates per treatment.

Hypoxic regulation of Dicer gene expression was initially measured at the protein level. Dicer protein levels in two different breast cancer cell lines (MCF7 and SKBR3 cells) exposed to hypoxia (0.1% O_2_) were examined by immunoblotting. After 16 h and 24 h of hypoxia (0.1% O_2_) no, or very modest, reductions in Dicer protein abundance were seen (see for example Figure 1A and Figure 1B). To accommodate the possibility that hypoxic alterations in Dicer levels were not seen at earlier time points because of significant stability of the Dicer protein, we examined the effects of longer durations of hypoxia. A striking reduction in Dicer protein levels was seen in both MCF7 (5 -fold) (Figure 1C) and SKBR3 cells (40-fold) (Figure 1D) when exposed to 0.1% O_2_ for 48 h, whilst the expression of the control gene alpha-actinin was unaffected by the hypoxic exposure. A striking reduction of Dicer protein levels (5.4 fold) was also observed even after a more modest hypoxic exposure (1% O_2_) for 72 h (Figure 1E).

### The effect of hypoxia on Dicer protein levels in human umbilical vein endothelial cells (HUVECs)

To examine whether hypoxic repression of Dicer was also observed in non cancer cells, human umbilical vein endothelial cells (HUVECs) were studied. HUVECs from multiple donors were exposed to different durations of hypoxia. A slight decrease in Dicer protein levels in HUVEC cells was seen after 1% O_2_ for 24 h (Figure 1F) and a more striking decrease (10-fold) after 1% O_2_ for 48 h (Figure 1G).

### The effect of hypoxia on Dicer mRNA levels in cancer cell lines

**Figure 2:**
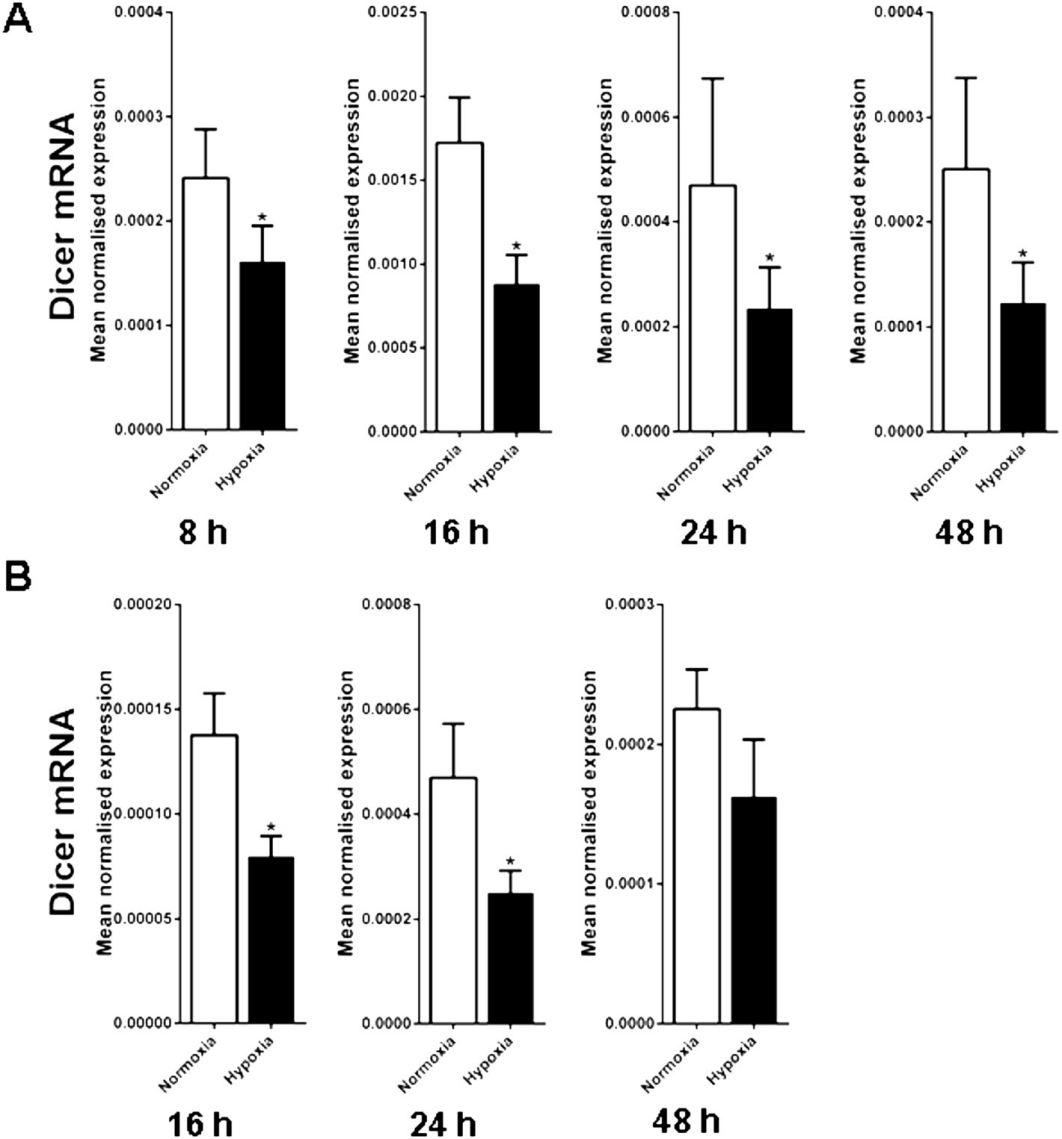
**Dicer mRNA expression in hypoxia vs. normoxia.** Dicer mRNA expression was examined in MCF7 and HT29 cells after exposure to 0.1% O_2_ for different durations. **A**, Dicer mRNA expression in MCF7 cells after exposure to 0.1% O_2_ for 8 h (P = 0.0007), 16 h (P = 0.0009), 24 h (P = 0.02) and 48 h (P = 0.008). **B**, Dicer mRNA expression in HT29 cells after exposure to 0.1% O_2_ for 16 h (P = 0.01), 24 h (P = 0.02) and 48 h (P = 0.09). *denotes P < 0.05 compared with parallel controls in normoxia. Data represent normalized mean ± S.E (error bars) (n = 3). Dicer mRNA levels were analysed by RT-PCR and normalised to 18S rRNA levels. Statistical significance established by Student’s t-test.

In previous array data we saw a significant and consistent repression of Dicer mRNA expression (data not shown). To examine whether the hypoxic repression of Dicer protein levels was regulated through repression of Dicer mRNA levels, we examined Dicer mRNA levels by RT PCR in MCF7 cells exposed to 0.1% O_2_ for 8, 16, 24 and 48 h, and observed significant down regulation of Dicer mRNA expression in hypoxia (Figure 2A). To examine if this repression was observed in other cancer cell lines we also examined the response of the colorectal cell line HT29 to varying durations of hypoxia and saw a similar, albeit less striking, reduction in Dicer mRNA levels (Figure 2B). However, no significant changes in Dicer mRNA levels were observed in SKBR3 breast cancer cells after being exposed to varying durations of hypoxia (24 h and 48 h, at 0.1% O_2_) despite seeing substantial reductions in Dicer protein expression.

### The mechanism of hypoxic repression of Dicer

#### The effect of HIF hydroxylase inhibitors on Dicer levels

**Figure 3:**
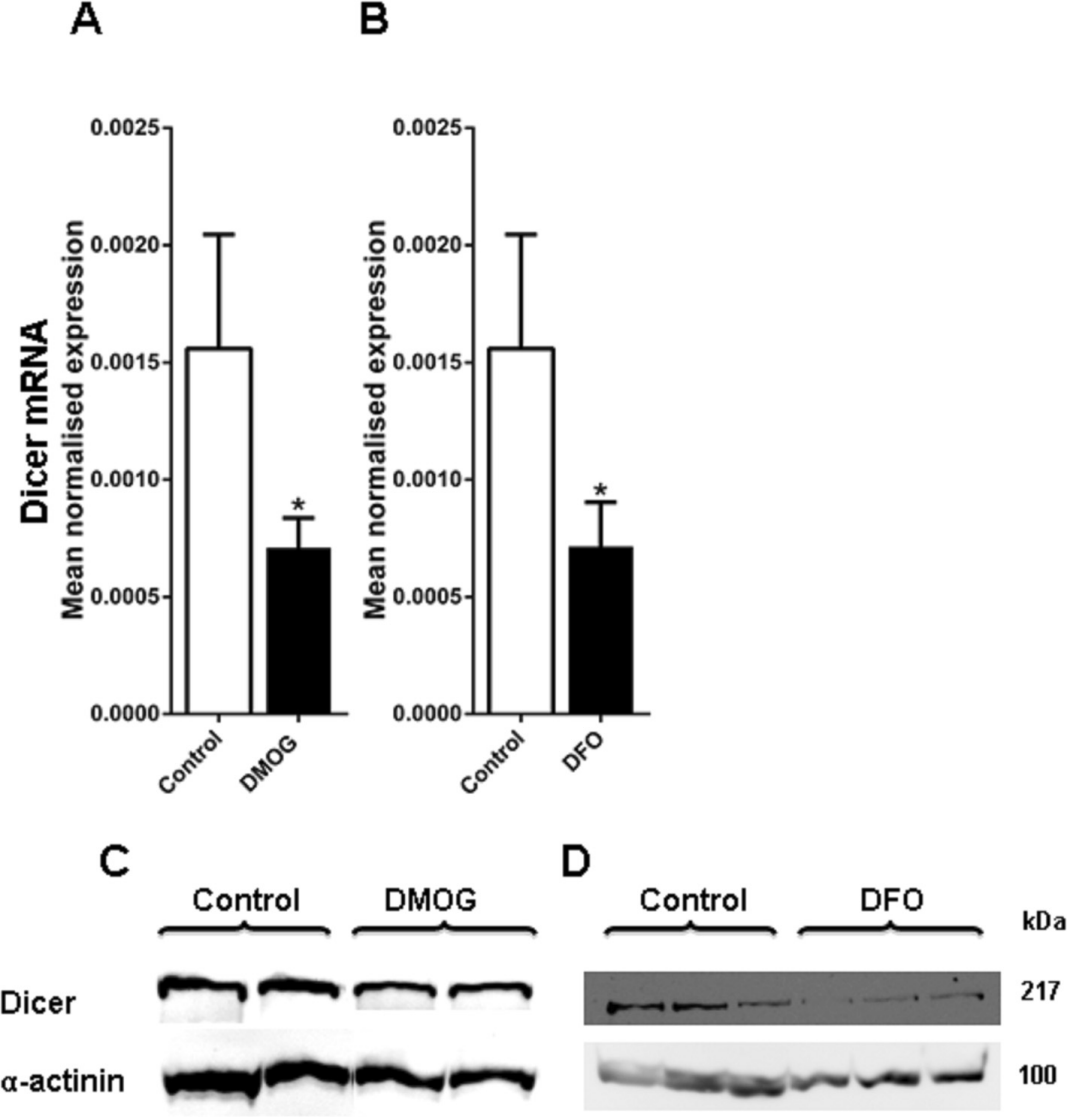
**Dicer mRNA and protein expression after exposure to HIF hydroxylase inhibitors.** Dicer mRNA and protein expression was examined in MCF7 and SKBR3 cells after exposure to HIF hydroxylase inhibitors: dimethyloxalyl glycine (DMOG) and desferrioxamine (DFO) **A**, Dicer mRNA expression (P = 0.04) in MCF7 cells after exposure to 1 mM DMOG for 48 h. **B**, Dicer mRNA expression in SKBR3 cells after exposure to 0.1 mM DFO for 48 h. *denotes P < 0.05 compared with parallel controls. Data represent normalized mean ± S.E (error bars) (n = 3). Dicer mRNA levels were analysed by RT-PCR and normalised to 18S rRNA levels. Statistical significance established by Student’s t-test. **C**, Dicer protein expression in MCF7 cells after exposure to 1 mM DMOG for 48 h Results show two technical replicates per treatment. **D**, Dicer protein expression in SKBR3 cells after exposure to DFO (0.1 mM) for 48 h. Results show three technical replicates per treatment. Dicer and α-actinin protein expression was examined by immunoblotting. α-actinin was used as the loading control.

HIF plays a central role in the transcriptional response to hypoxia, so the role of the HIF pathway in the hypoxic repression of Dicer levels was examined. Cells were exposed to the HIF hydroxylase inhibitors, dimethyloxalyl glycine (DMOG) and desferrioxamine which induce HIF levels under normoxic conditions [36,39]. A modest decrease in Dicer mRNA levels was seen after exposing MCF7 cells to DMOG (1 mM) for 48 h (Figure 3A) and desferrioxamine (0.1 mM) for 48 h (Figure 3B). A similar influence on Dicer protein levels was seen following exposure to HIF hydroxylase inhibitors. A modest decrease in Dicer protein levels was seen in MCF7 cells after exposure to DMOG (1 mM) for 48 h (Figure 3C). A more substantial decrease in Dicer protein levels were seen in SKBR3 cells after exposure to desferrioxamine (0.1 mM) for 48 h (Figure 3D).

### Involvement of HIF in Dicer regulation

**Figure 4:**
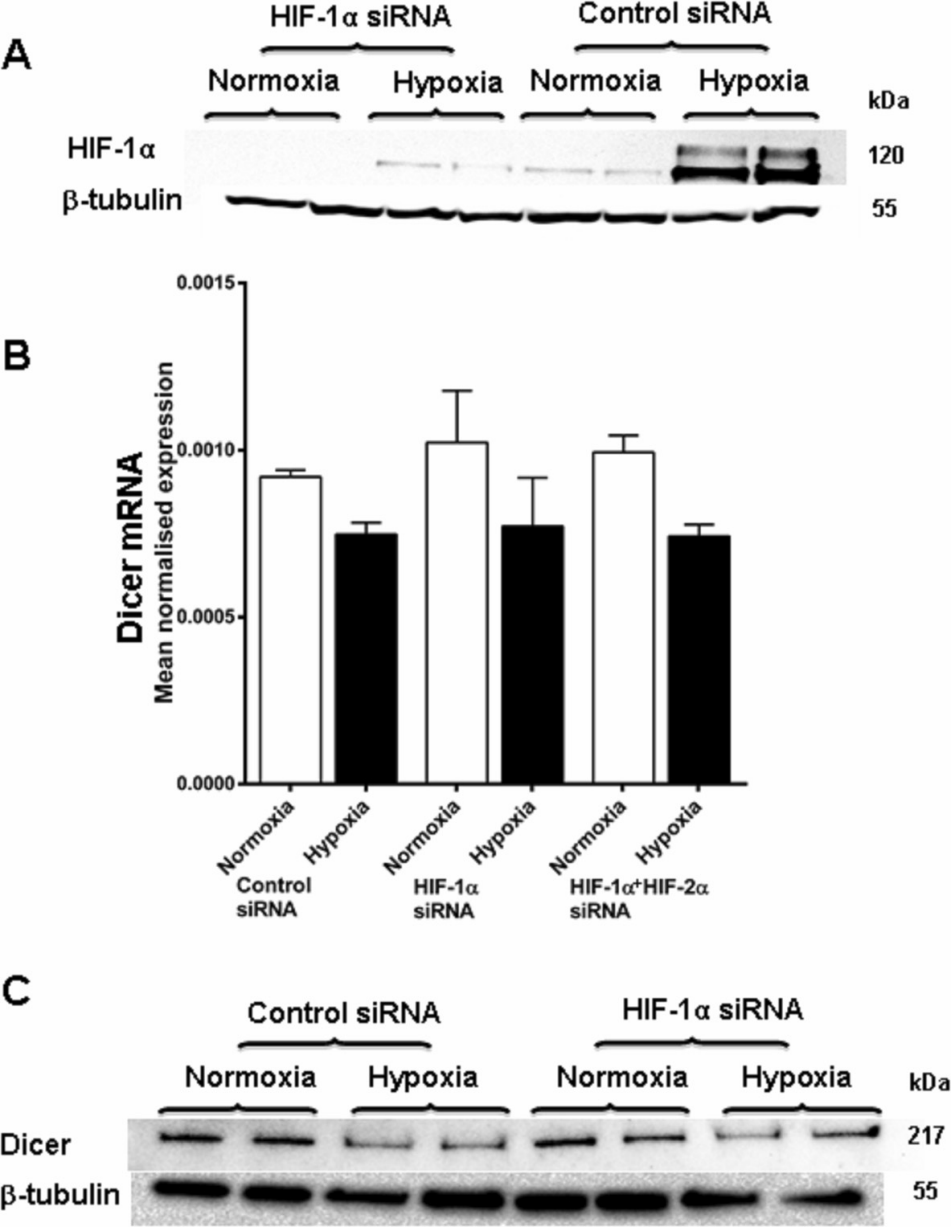
**Dicer repression in hypoxia is not HIF dependent.** Dicer expression was examined after HIF-1α and HIF-2α inhibition with siRNA in hypoxia compared to normoxia **A**, HIF-1α protein expression in SKBR3 cells after transient transfection with a HIF-1α targeting siRNA or control siRNA, then exposure to hypoxia (0.1% O_2_ for 24 h) vs. normoxia. Results show two technical replicates per treatment. **B**, Dicer mRNA expression in MCF7 cells after transient transfection with HIF-1α, HIF-2α or control siRNA, then exposure to hypoxia (0.1% O_2_ for 48 h) vs. normoxia. Data represent normalized mean ± S.E (error bars) (n = 3). Dicer mRNA levels were analysed by RT q-PCR and normalised to β-2-microglobulin mRNA levels. **C**, Dicer protein expression in SKBR3 cells after transient transfection with HIF-1α targeting siRNA or control siRNA, then exposure to hypoxia (0.1% O_2_ for 48 h) vs normoxia. Results show two technical replicates per treatment. Dicer and α-actinin protein levels were examined by immunoblotting. α-actinin was used as the loading control.

To further investigate the involvement of HIF in Dicer regulation RNA interference was used to inhibit HIF-1α and HIF-2α, and Dicer levels was examined. The HIF-1α RNA interference substantially reduced the hypoxic expression of HIF-1α protein (Figure 4A). The influence of HIF-1α and HIF-2α isoforms in hypoxic repression of Dicer was examined. When HIF-1α and HIF-2α were inhibited simultaneously using RNA interference there was no effect on the *DICER* mRNA repression, suggesting a lack of HIF-1α or HIF-2α mediated down regulation of *DICER* mRNA levels after exposure to hypoxia (Figure 4B). Substantial repression of Dicer protein was observed under hypoxia but there was no significant alteration in hypoxic repression of Dicer protein after HIF-1α RNA interference (Figure 4C).

### Prolyl hydroxylase dependent regulation of Dicer

**Figure 5:**
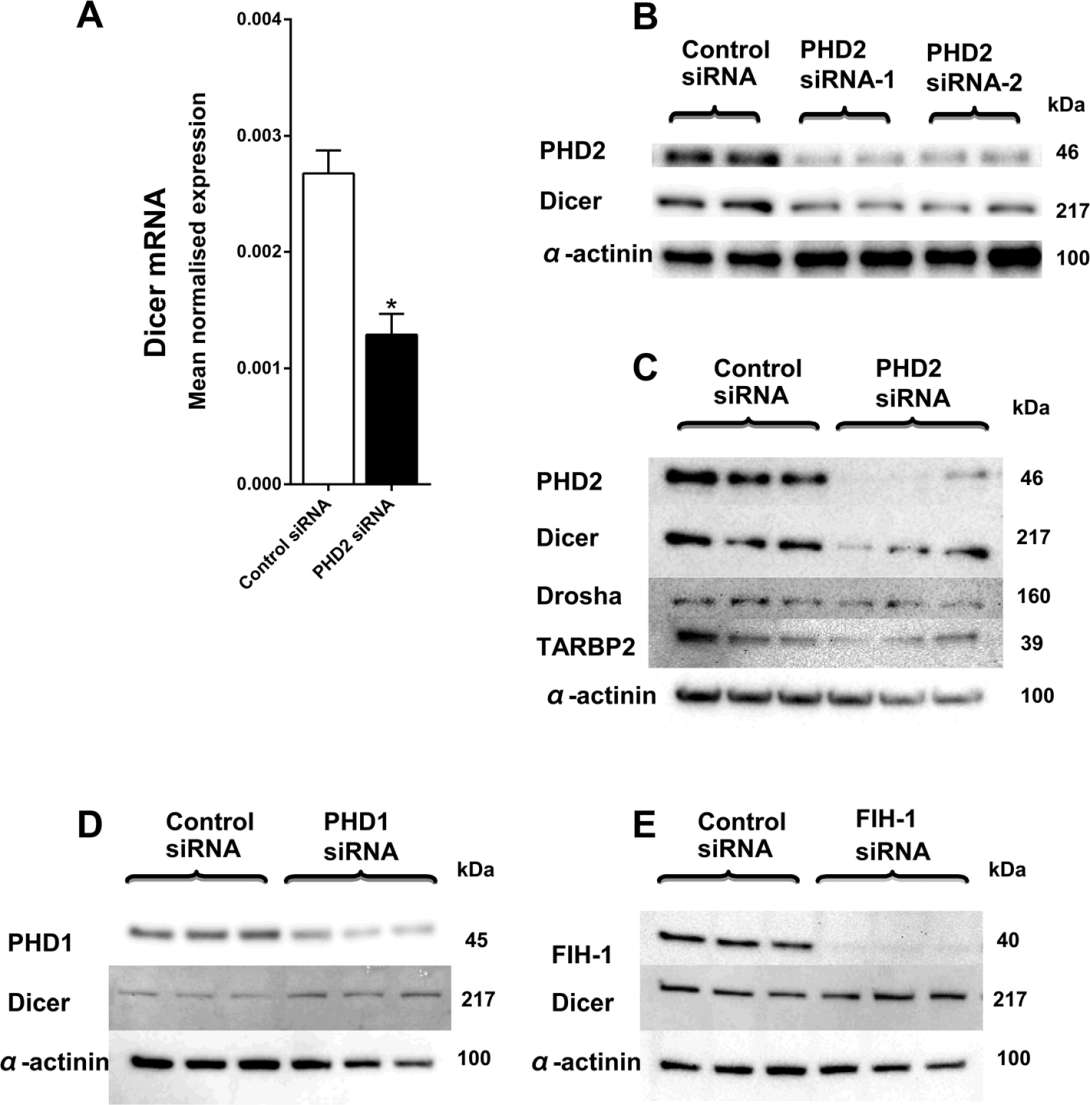
**Prolyl hydroxylase dependent regulation of Dicer.** Dicer expression was examined in SKBR3 cells after inhibition of each prolyl hydroxylase by siRNAs. **A**, Dicer mRNA expression in SKBR3 cells after transient transfection with PHD2 targeting siRNAs or control siRNA in normoxia (P = 0.0008). *denotes P < 0.05 compared with parallel controls. Data represent normalized mean ± S.E (error bars) (n = 3). Dicer mRNA levels were analysed by RT-PCR and normalised to β-2-microglobulin mRNA levels. Statistical significance established by Student’s t-test. **B**, PHD2 and Dicer protein levels after transient transfection of SKBR3 cells with two independent siRNAs targeting PHD2 or control siRNAs in normoxia. Results show two technical replicates per treatment. **C**, Dicer, Drosha and TARBP2 protein expression after transient transfection of SKBR3 cells with siRNAs targeting PHD2 or control siRNAs in normoxia. **D**, Dicer protein expression after transient transfection of SKBR3 cells with siRNAs targeting PHD1 siRNAs or control siRNAs in normoxia. **E**, Dicer protein expression after transient transfection of SKBR3 cells with siRNAs targeting FIH-1 siRNA or control siRNA in normoxia. Results show three technical replicates per treatment. Protein expression was examined by immunoblotting. α-actinin was used as the loading control.

These earlier results pointed towards a mechanism of regulation that was oxygen and HIF hydroxylase dependent but was independent of HIF-1α. To investigate this we used siRNA mediated suppression of PHD2 expression in SKBR3 cells and determined the levels of Dicer. A significant decrease in Dicer mRNA (P = 0.0008) (Figure 5A) and a large decrease in Dicer protein (Figure 5B) levels, were evident following PHD2 suppression in normoxia. This observation was validated by repeating the experiment with a different PHD2 siRNA and a similar effect was seen on Dicer expression. PHD2 suppression did not affect Drosha and TARBP2 protein levels (Figure 5C). We also examined if the other HIF hydroxylase enzymes (PHDs or Factor Inhibiting HIF-1 (FIH-1)) were involved in Dicer regulation by hypoxia. In contrast to the effects of PHD2 suppression, there was no decrease in Dicer expression after PHD1 (Figure 5D) or FIH-1 RNA interference (Figure 5E). In keeping with previous work, PHD3 levels were very low under normoxic conditions [40].

### The involvement of microRNAs in Dicer regulation by hypoxia

**Figure 6:**
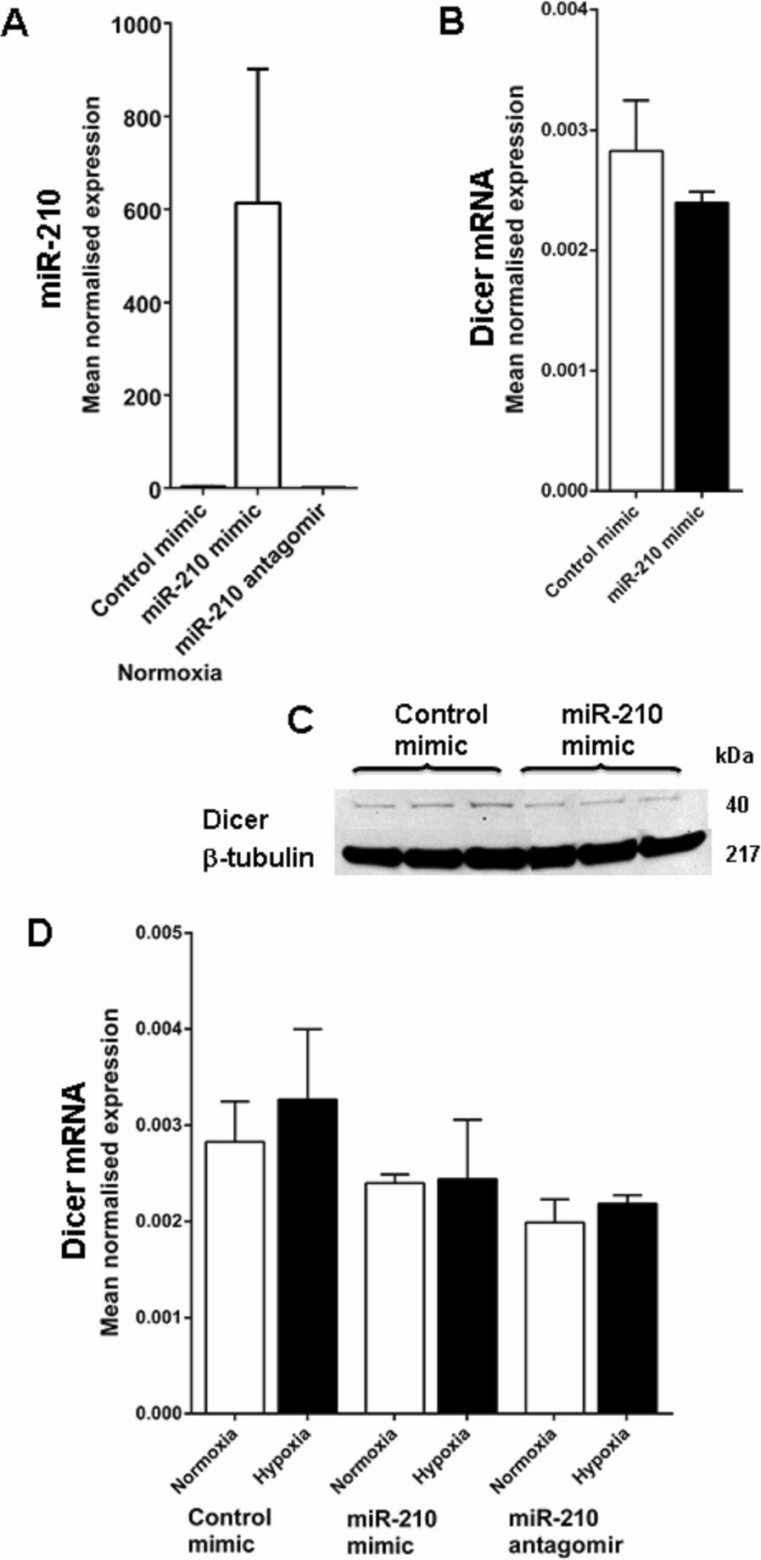
**Dicer expression in not regulated by miR-210 in hypoxia.** Dicer expression was examined after transient transfection with a miR-210 mimic and miR-210 antagomir in SKBR3 cells. **A**, miR-210 expression in SKBR3 cells after transient transfection with control mimic and miR-210 mimic and miR-210 antagomir in normoxia. **B**, Dicer mRNA expression in SKBR3 cells after transient transfection with control mimic and miR-210 mimic. **C**, Dicer protein expression in SKBR3 cells after transfection with control mimic and miR-210 mimic. Results show three technical replicates per treatment. **D**, Dicer mRNA expression in SKBR3 cells after transient transfection with control mimic, miR-210 mimic and miR-210 inhibitor, then exposed to 1% O_2_ for 72 h. Data represent normalized mean ± S.E (error bars) (n = 3). mRNA levels were analysed by RT-PCR and normalised to β-2-microglobulin mRNA levels. Statistical significance established by Student’s t-test. Dicer and β-tubulin protein levels were examined by immunoblotting. β-tubulin was used as the loading control.

In other experimental settings, the feedback regulation of Dicer by specific Dicer dependent miRNAs has been reported to be important in controlling Dicer levels [41,42]. We therefore hypothesised that a hypoxically induced miRNA might mediate the hypoxic repression of Dicer expression. miR-210 is the best characterised example of a miRNA that shows substantial induction under hypoxic conditions. We examined the influence of miR-210 manipulations on Dicer expression. miR-210 was over-expressed in normoxia by transient transfection of SKBR3 cells with a miR-210 mimic and a control mimic (Figure 6A). We determined Dicer mRNA (Figure 6B) and protein (Figure 6C) levels after over-expressing miR-210. Only a slight decrease in Dicer mRNA levels was observed with miR-210 over-expression, and Dicer levels were not affected by the miR-210 antagomir in normoxic or hypoxic conditions (Figure 6D).

**Figure 7:**
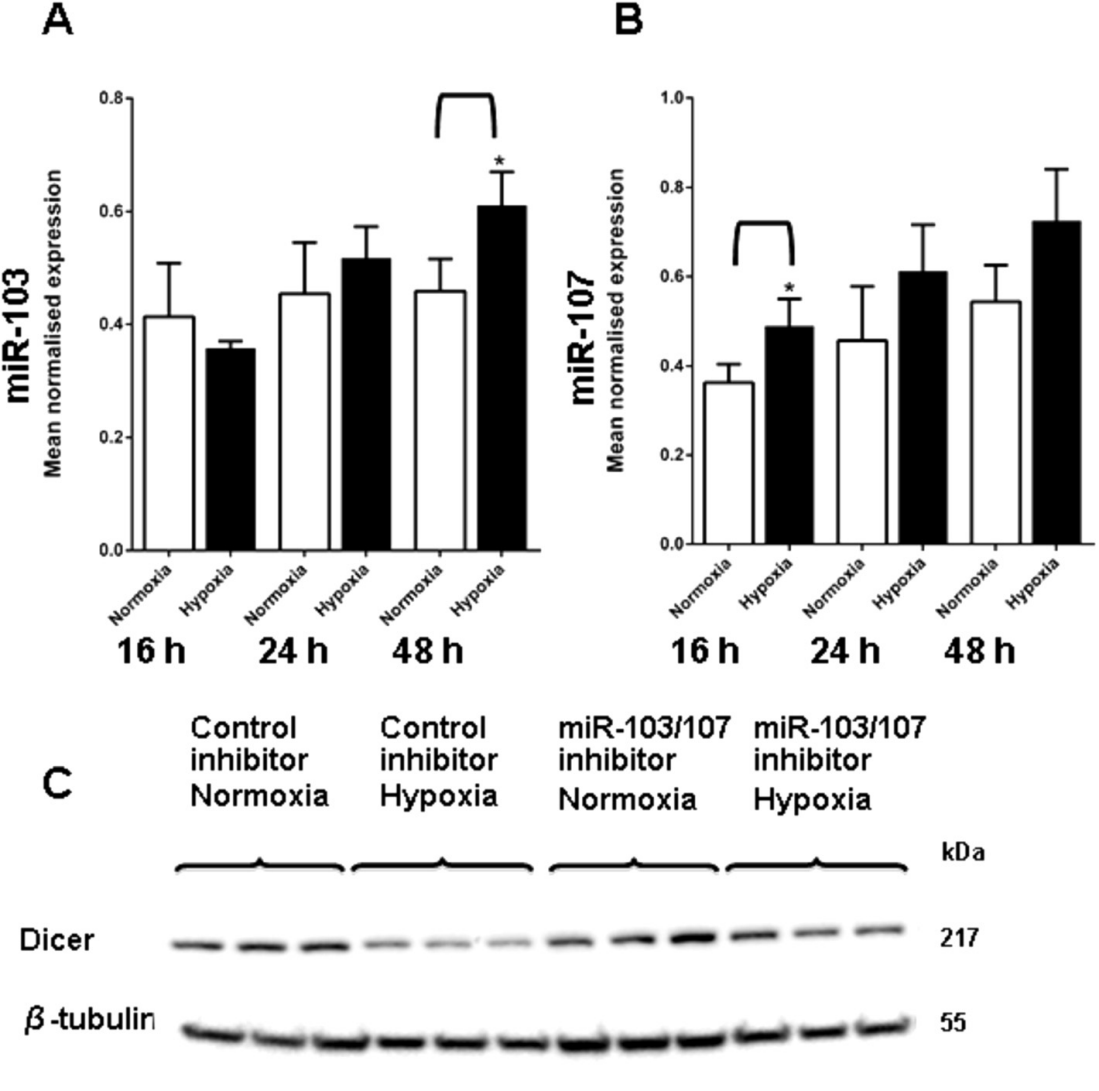
**Dicer repression by miR-103 and miR-107 in hypoxia.** Expression of miR-103 and miR-107 were examined in MCF7 cells, after exposure to 0.1% O_2_ for different durations. **A**, miR-103 expression in MCF7 cells after hypoxia (0.1% O_2_ for 16, 24 and 48 h) vs. normoxia (P = 0.03). **B**, miR-107 expression in MCF7 cells after hypoxia (0.1% O_2_ for 16, 24 and 48 h) vs. normoxia (P = 0.04). * denotes p < 0.05 compared with parallel controls. Data represent normalized mean ± S.E (error bars) (n = 3). miRNA levels were analysed by q-RT PCR and normalised to U6 levels. Statistical significance established by Student’s t-test. Dicer expression was examined after transient transfection with miR-103/107 inhibitors and exposure to hypoxia vs. normoxia. **C**, Dicer protein expression after transfection of MCF7 cells with miR-103/107 inhibitors or control inhibitors and exposure to hypoxia (0.1% O_2_ for 48 h) vs*.* normoxia. Results show three technical replicates per treatment. Dicer and α-actinin protein levels were examined by immunoblotting. α-actinin was used as the loading control.

To further test possible miRNA involvement in Dicer regulation, we examined for the hypoxic regulation of miRNAs with well characterised roles in Dicer regulation. Martello et al., (2010) found conserved binding sites for miR-103/107 family in the 3’UTR of *DICER*. With the aid of luciferase reporter constructs containing the wild type or mutant miR-103/107 binding sites they showed that Dicer is regulated by these two miRNAs. In addition, miR-103 and miR-107 have also been identified to decrease miRNA biogenesis by targeting Dicer in cancer [41] and have been reported to show hypoxic induction in some situations [32]. We examined miR-103 and miR-107 levels in MCF7 cells after exposure to hypoxia (0.1% O_2_ for 16, 24 and 48 h) vs. normoxia to see if these miRNAs were affected by hypoxia. There was a modest increase in miR-103 levels after 24 h of hypoxia (0.1% O_2_) and a more significant increase after 48 h of hypoxia (Figure 7A). There was an increase in miR-107 levels after 16 h of hypoxia (0.1% O_2_) and at 24 and 48 h (Figure 7B). To examine if the down regulation of Dicer levels in hypoxia was regulated by these two miRNAs, the effects of miR-103/107 antagomirs was tested on Dicer protein expression under hypoxia and normoxia by immunoblotting. In keeping with a role for miR-103/107 in the hypoxic repression of Dicer expression, the blocking of miR-103/107 function abrogated the hypoxic repression of Dicer levels (Figure 7C).

### Hypoxic regulation of other miRNA biogenesis proteins

**Figure 8:**
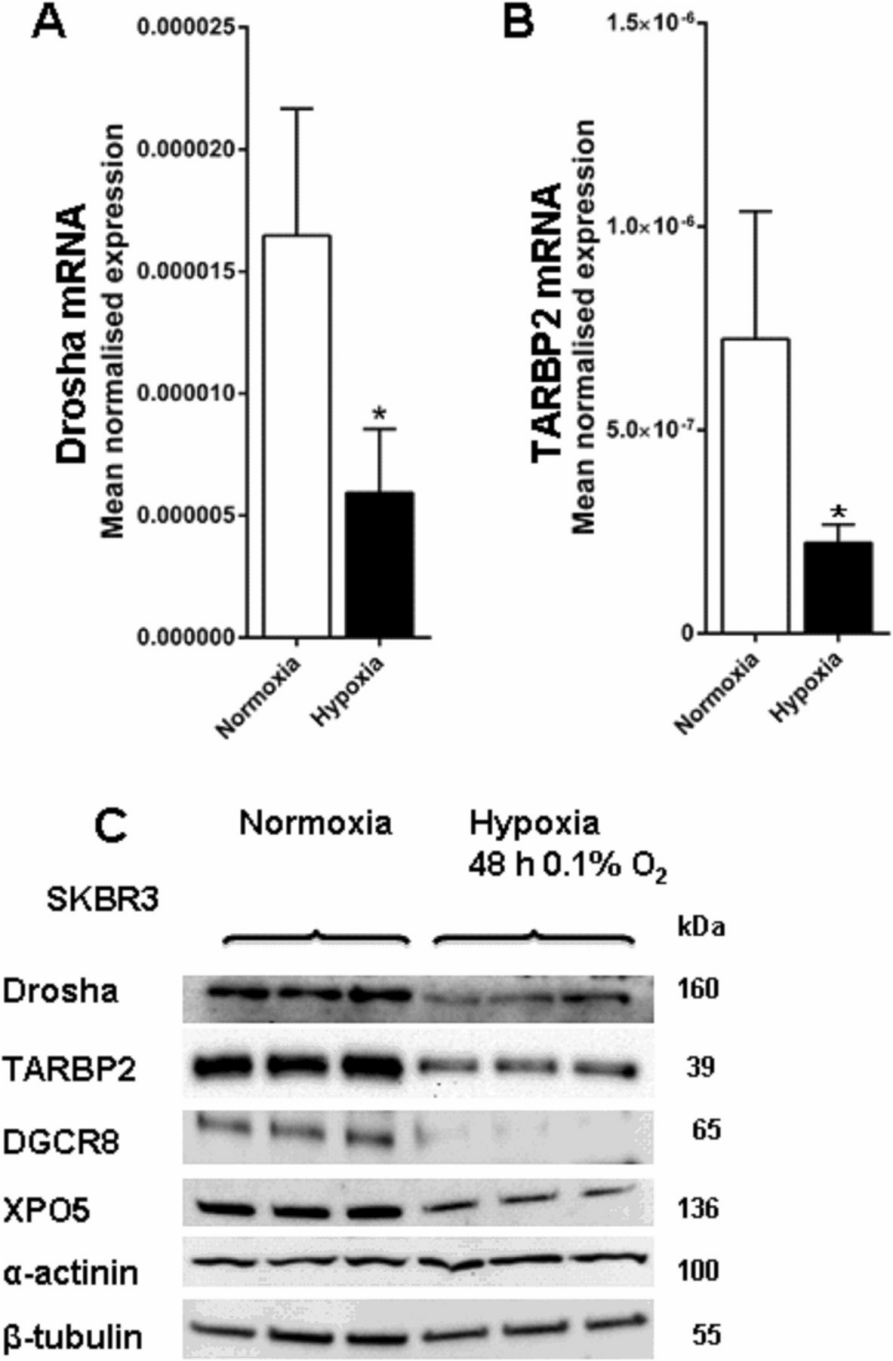
**Hypoxic regulation of other miRNA biogenesis proteins.** Expression of miRNA biogenesis proteins Drosha, TARBP2, DGCR8 and XPO5 were examined under hypoxia vs. normoxia **A**, Drosha mRNA expression in SKBR3 cells after hypoxia (0.1% O_2_ 48 h) vs*.* normoxia (P = 0.05). **B**, TARBP2 mRNA expression in SKBR3 cells after hypoxia (0.1% O_2_ 48 h) vs*.* normoxia (P = 0.03). *denotes P < 0.05 compared with parallel controls. Data represent normalized mean ± S.E (error bars) (n = 3). mRNA levels were analysed by RT-PCR and normalised to 18S rRNA levels. **C**, Drosha, TARBP2, DGCR8 and XPO5 protein expression in SKBR3 cells after hypoxia (0.1% O_2_ 48 h) vs*.* normoxia. Results show three technical replicates per treatment. Protein levels were examined by immunoblotting. α-actinin and β-tubulin used as the loading controls.

The hypoxic regulation of other proteins involved in the miRNA biogenesis process was also examined. A consistent repression of Drosha (Figure 8A) and TARBP2 (Figure 8B) mRNA expression, measured by RT-PCR, was observed in MCF7 cells exposed to 0.1% O_2_ for 48 h. Protein levels were also examined after exposure to 0.1% O_2_ for 48 h, and there was a striking down regulation of Drosha, TARBP2, DGCR8 and XPO5 protein levels in hypoxia (Figure 8C). The control protein levels (α-actinin and β-tubulin) did not change following these severities and durations of hypoxic exposures.

### Co-ordinated expression of miRNA biogenesis proteins

Given the hypoxic down regulation of many proteins involved in miRNA biogenesis, the mechanisms responsible for this effect was examined. Previously others had shown a strong correlation between Dicer protein and TARBP2 protein levels [11,43]. To investigate possible co-ordinated control of protein levels, specific RNA interference was used to inhibit the expression of each gene and then the levels of the other proteins were analysed by immunoblotting.

**Figure 9:**
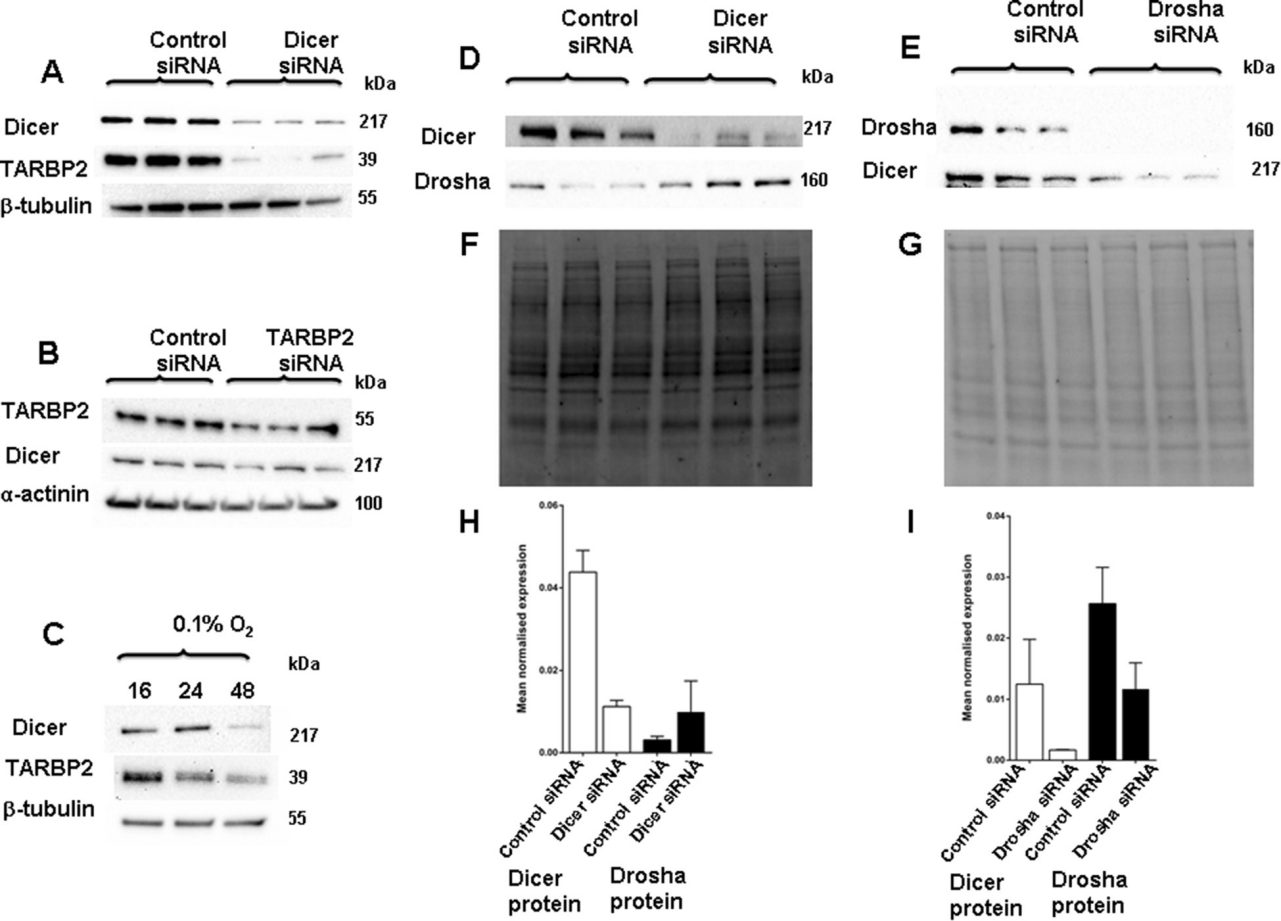
**Co-ordinated expression of miRNA biogenesis proteins. A**, Dicer and TARBP2 protein expression in SKBR3 cells after transient transfection with siRNA targeting Dicer or control siRNA. Results show three technical replicates per treatment. **B**, TARBP2 and Dicer protein expression in SKBR3 cells after transient transfection with siRNA targeting TARBP2 or control siRNA. **C**, Changes in Dicer and TARBP2 protein expression after exposure to 16, 24 and 48 h of hypoxia. **D**, Dicer and Drosha protein expression in SKBR3 cells after transient transfection with siRNA targeting Dicer or control siRNA. Results show three technical replicates per treatment. **E**, Dicer and Drosha protein expression in SKBR3 cells after transient transfection with siRNA targeting Drosha or control siRNA. Results show three technical replicates per treatment. **F** and **G**, total protein on PVDF membranes. **H** and **I**, Densitometric analysis of **D** and **E** following normalisation with total protein on PVDF membrane. Protein levels were examined by immunoblotting. α-actinin, β-tubulin or total protein on the PVDF membrane were used as the loading controls.

When Dicer was inhibited using RNA interference a large decrease in TARBP2 levels (Figure 9A) was observed, in keeping with previous reports [43]. Similarly, when TARBP2 levels were reduced by RNA interference in SKBR3 cells there was a modest decrease in Dicer protein expression (Figure 9B). When Dicer and TARBP2 levels were studied over a time course both proteins seemed to decrease at the same rate (Figure 9C). A relationship between Dicer and Drosha levels was also observed with an increase in Drosha protein expression after inhibiting Dicer (Figure 9D) while a decrease in Dicer protein levels was seen after Drosha inhibition by siRNA treatment (Figure 9E).

### Mature and precursor microRNA levels in hypoxia

The previous results indicated that hypoxia down regulates proteins involved in the miRNA biogenesis pathway. For a more comprehensive understanding of the global effects of hypoxia vs. normoxia on miRNA biogenesis, mature and precursor miRNA levels were compared using Affymetrix miRNA 3.1 arrays. Mature and precursor miRNA levels in MCF7 cells exposed to hypoxia (0.1% O_2_ for 16 h) or normoxia were analysed. In keeping with the modest changes in Dicer expression at this time point, no significant changes were observed in mature or precursor miRNA levels in MCF7 cells exposed to hypoxia (0.1% O_2_) vs. normoxia after 16 h (data not shown).

In a similar experiment undertaken with a longer duration of hypoxic exposure (0.1% O_2_ for 48 h) several miRNAs were significantly up or down regulated in MCF7 cells (see Additional file 1: Figure S1). Eight miRNAs were significantly up regulated (see Additional file 1: Table S2), and four miRNAs were significantly down regulated in hypoxia when compared to normoxia (see Additional file 1: Table S3). Even following a longer duration of hypoxic exposure (0.1% O_2_ for 48 h) there were no significant changes between individual precursor miRNA: mature miRNA ratios either with hypoxia (see Additional file 1: Figure S2) or, surprisingly, following Dicer suppression by siRNA (see Additional file 1: Figure S3-S4). The microarray data discussed in this publication have been deposited in NCBI’s Gene Expression Omnibus [38] and are accessible through GEO Series accession number GSE49999.

**Figure 10:**
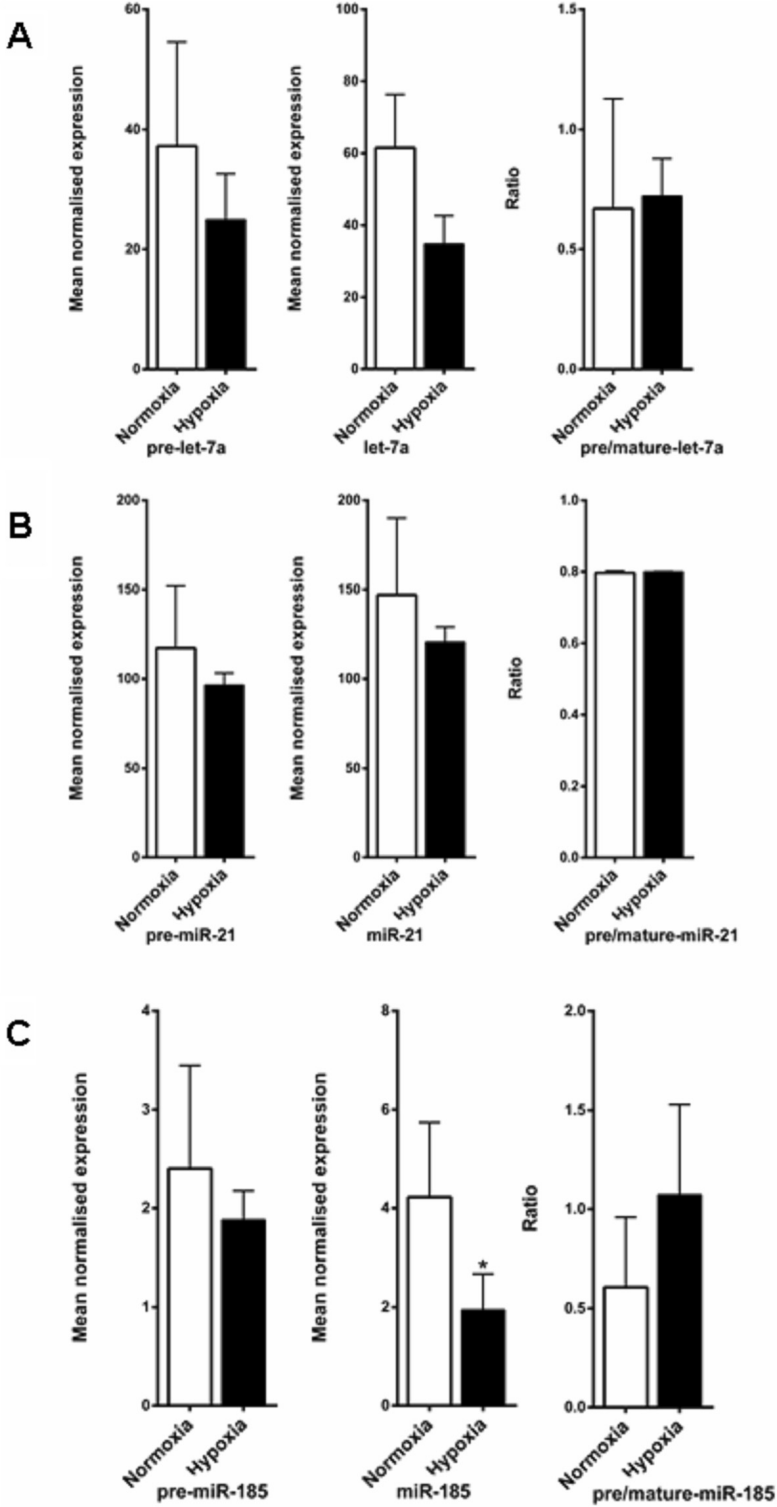
**Mature and precursor expression in hypoxia vs. normoxia. A**, Pre-let-7a and mature let-7a expression, precursor/mature ratio in MCF7 cells exposed to hypoxia (0.1% O_2_ for 48 h) vs. normoxia. **B**, Pre-miR-21 and miR-21 expression, precursor/mature ratio in MCF7 cells exposed to hypoxia (0.1% O_2_ for 48 h) vs. normoxia**. C**, Pre-miR-185 and miR-185 expression, precursor/mature ratio in MCF7 cells exposed to hypoxia (0.1% O_2_ for 48 h) vs. normoxia. Data represent normalized mean ± S.E (error bars) (n = 3). miRNA levels were analysed by RT-PCR and normalised to RNU6B levels.

To explore the effects of hypoxia on the processing of specific miRNAs with a different assay, the levels of mature and precursor miRNA levels of let-7a, miR-21 and miR-185 were determined in MCF7 cells exposed to hypoxia vs. normoxia, by RT-PCR. These were selected as previous reports showed they were Dicer dependent miRNAs [44-46]. Only a modest decrease in mature and precursor levels of let-7a and miR-21 in hypoxia was observed, and no accumulation of pre-let-7a or pre-miR-21 in hypoxia was evident (Figure 10A, 10B). A significant reduction (P = 0.03) in mature miR-185 was observed in hypoxia in MCF7 cells (Figure 10C), but no accumulation of precursors was seen. Furthermore following the recent report of hypoxia reducing miRNA processing by Ho et al. [45] in HUVEC cells, the ratio of mature and pre-miRNA levels for miR-185 and miR-21 was examined in these cells. However, following both 24 and 48 h of 1% hypoxia again there was no clear effect on processing (see Additional file 1: Figure S4-S5). Therefore, only a minimal overall effect on miRNA processing was observed for these specific miRNAs, consistent with the lack of effect on processing as seen in the microarray experiment.

### The effect of hypoxia on microRNA function

**Figure 11:**
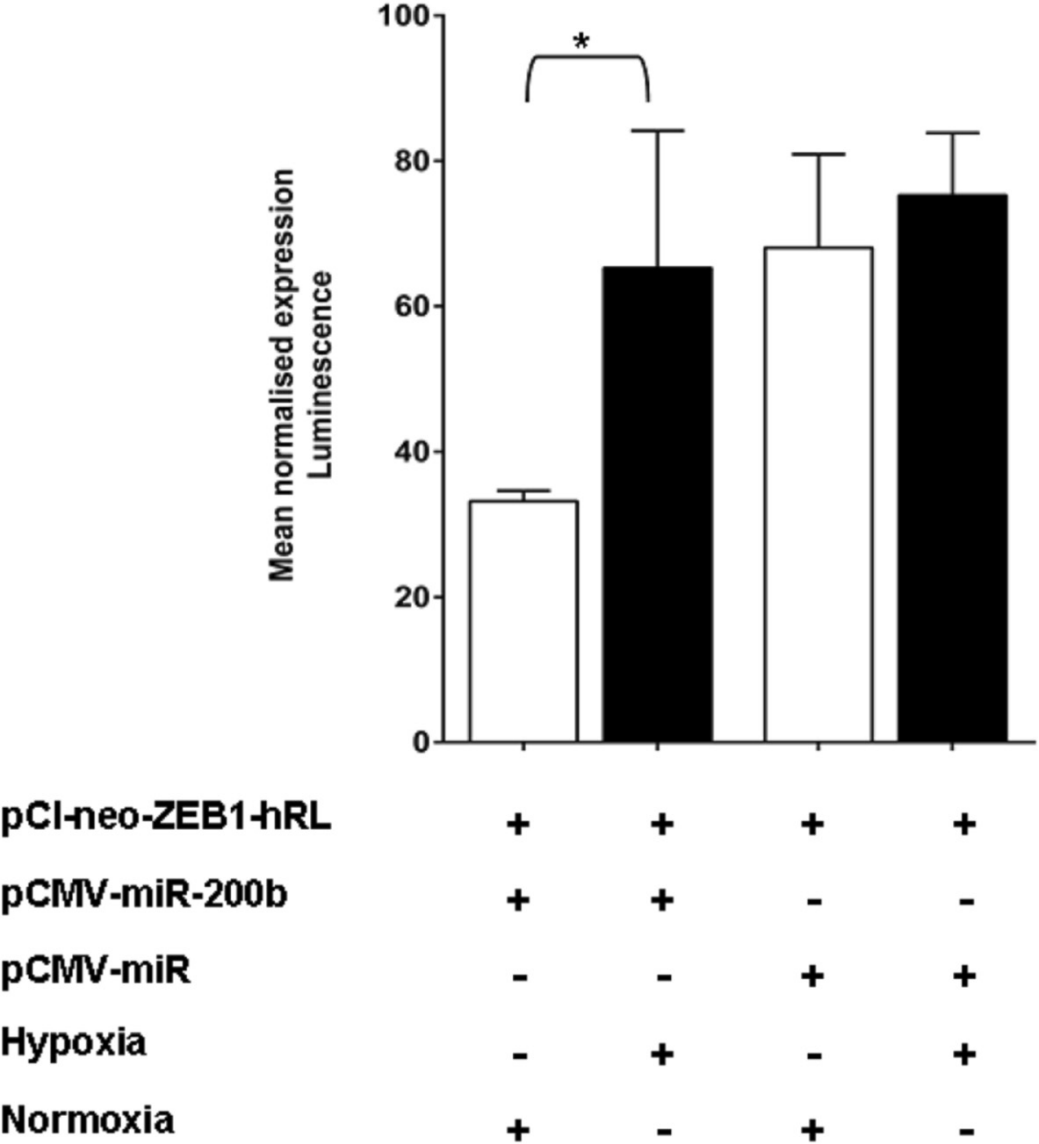
**miRNA processing is affected by hypoxia.** Luciferase activity in SKBR3 cells after co-transfection with RL reporter containing a ZEB1 WT with miR-200b target sites (pCi-neo-ZEB1-hRL) and precursor miR-200b (pCMV-miR-200b) or empty vector (pCMV-miR) (Origene), then exposure to hypoxia (0.1% O_2_ 24 h) *vs*. normoxia (P = 0.04). *denotes P < 0.05 compared with parallel controls. Data represent normalized mean ± S.E (error bars) from two independent experiments consisting of three technical replicates.

Given the multiple potential influences of hypoxia on miRNA biogenesis proteins and RISC activity, the effects of hypoxia on miRNA function were studied. For this a plasmid containing the 3’UTR of ZEB1 gene downstream of a Renilla luciferase (RL) reporter was used [37] along with a plasmid that expresses a pre-miR-200b. ZEB1 is an E-cadherin transcriptional repressor involved in epithelial to mesenchymal transition of tissues and is regulated by the miR-200 family. SKBR3 cells were co-transfected with a plasmid containing the 3’UTR from the ZEB1 gene downstream of a Renilla luciferase reporter (pCi-neo-ZEB1-hRL) together with precursor miR-200b (pCMV-miR-200b) or empty vector (pCMV-miR), then cells were exposed to hypoxia (0.1% O_2_ for 24 h) or normoxia, and luciferase activity was measured. ZEB1 3’UTR contains miR-200b target sites and binding of the mature miR-200b will repress the RL activity. The luminescence measured with pCi-neo-ZEB1-hRL + pCMV-miR-200b showed a significant repression in normoxia when compared to hypoxia, suggesting less miR-200b mediated suppression in hypoxia (Figure 11). No change in luminescence was seen in the control (pCi-neo-ZEB1-hRL + pCMV-miR) transfection after incubation in hypoxia compared with normoxia (Figure 11). Results show data from two independent experiments each with three technical replicates.

## Discussion

This study examined the hypoxic regulation of miRNA biogenesis proteins in cancer cells. Results showed that hypoxia down regulates the levels of Dicer and several other proteins (Drosha, TARBP2, DGCR8 and XPO5) in]volved in miRNA biogenesis. The hypoxic down regulation of Dicer protein levels was observed in multiple cell lines, suggesting that this could be a common phenomenon and not restricted to cancer cells. The reductions in Dicer protein levels were much more significant following longer durations of hypoxia (for 48 and 72 h) and with greater severity of hypoxic exposure (0.1% O_2_). One likely explanation for this observation is high stability of Dicer protein. Therefore, the decrease in transcription might not be readily evident until 48 h. Consistent with this explanation, only modest reductions in Dicer protein levels were observed at shorter durations of hypoxic exposure. The mechanism of this hypoxic regulation appears to operate at several levels. The hypoxic suppression of Dicer mRNA levels appears to account for regulation in some (MCF7 and HT29) but not all cells (e.g. SKBR3) indicating the operation of other mechanisms in the hypoxic regulation of Dicer. During the course of this work, hypoxic repression of Dicer mRNA and protein was also reported by others [34,45,47]. In exploring the mechanism of hypoxic regulation, we investigated the role of HIF and the HIF hydroxylases. In keeping with a role for the HIF hydroxylases in mediating this hypoxic repression, when cells were exposed to the HIF hydroxylase inhibitors, DMOG and desferrioxamine, a modest decrease in Dicer mRNA and protein levels was observed in MCF7 cells. However, somewhat surprisingly no, or very modest, effects on the repression of Dicer mRNA and protein levels were observed after HIF-1α and HIF-2α inhibition using siRNA treatments. This suggested that HIF did not play a major role in this hypoxic regulation of Dicer, a conclusion also reached by Ho et al. (2012) [45], However, in a recent study Camps, et al. (2014) reported down regulation of Dicer mRNA levels in hypoxia in a HIF dependent manner [48].

Our results indicate a mechanism of regulation that is oxygen and HIF hydroxylase dependent but independent of HIF. To explore this mechanism further, we examined for the possible contribution of each of the three HIF prolyl hydroxylases (PHD1, 2 and 3) and the HIF asparaginyl hydroxylase, FIH-1. When PHD2 was suppressed by RNA interference in normoxic conditions significant decreases in Dicer mRNA and protein expression were observed in normoxia, indicating a role for PHD2 in this hypoxic regulation. Interestingly, a recent study described a PHD2 dependent, but HIF independent, pathway for repressing protein synthesis in hypoxia. They reported of a PHD2 mediated phosphorylation of the eukaryotic elongation factor-2 and inhibition of protein translation under acute hypoxia [49]. In keeping with such a mechanism, the other HIF hydroxylase enzymes (PHD1, PHD3) and FIH-1 were not involved in Dicer regulation by hypoxia.

In further exploring the mechanisms of hypoxic regulation and given the importance of miRNA mediated feedback loops in control of Dicer expression [41,42], we examined possible influence of hypoxically induced miRNAs in mediating hypoxic repression of Dicer expression. Hypoxically induced miR-210 is the best characterised example of a miRNA that shows substantial induction under hypoxic conditions [32,50] but had no significant influence on the levels of Dicer protein.

Two miRNAs, miR-103 and miR-107 have been shown to decrease miRNA biogenesis by targeting Dicer in cancer [41] and are reportedly induced by hypoxia in some situations [32]. To examine their potential contribution to hypoxic repression of Dicer, cells were exposed to hypoxia after inhibiting miR-103 and miR-107 with antagomirs. The repression of Dicer mRNA and protein levels in hypoxia was largely abrogated, consistent with a role for miR-103 and miR-107 in the hypoxic regulation of Dicer.

Other proteins involved in miRNA biogenesis (Drosha, TARBP2, DGCR8 and XPO5) also showed significant down regulation under hypoxia when compared to normoxia. A previous microarray study also showed modest but consistent hypoxic reductions in mRNA levels of genes (Dicer, TARBP2 and AGO2) involved in miRNA biogenesis [36]. Ho *et al*. (2012) also reported a decrease in DGCR8 and XPO5 protein levels in hypoxia, (though they did not see a decrease in Drosha levels in hypoxia) [45]. This suggests the operation of a broader influence of hypoxia on the expression of genes encoding proteins that are essential in miRNA biogenesis. This is consistent with previous work indicating co-ordinate regulation of the levels of these proteins. When Dicer expression was suppressed there was a significant decrease in TARBP2 levels in keeping with previous reports [43]. Similarly, when TARBP2 levels were reduced by siRNA treatment there was a modest decrease in Dicer protein. When Dicer and TARBP2 levels were examined over a time course of hypoxic exposure both proteins seemed to decrease co-ordinately. Previously others have shown a powerful influence of the levels of Dicer protein on the levels of TARBP2 and vice versa [11,43] and post transcriptional cross regulation between Drosha and DGCR8 [51]. In this work we have also observed a further relationship linking Dicer and Drosha expression. Recent work has shown that Argonaute 2 stability is dependent on the availability of mature miRNAs. Dicer knockout influenced the mature miRNA production leading to decreased AGO2 stability [52]. Similar mechanisms might be operating to co-ordinate the levels of other miRNA biogenesis proteins in hypoxia.

Even though there was a significant and consistent reduction in the levels of proteins with central roles in miRNA biogenesis machinery under hypoxia, we did not see a substantial effect of this on the expression levels of mature miRNAs over the time course of these experiments. Indeed under these conditions Dicer suppression by RNA interference was only associated with slight alterations in mature miRNA abundance. This was true both for miRNAs assessed by microarray studies and also when we focussed on examining the levels of particular miRNAs and their precursors by RT-PCR. Microarray analysis of MCF7 cells exposed to hypoxia showed eight miRNAs were significantly up regulated and four miRNAs were significantly down regulated. Of the eight up regulated miRNAs, miR-210 has been well validated as a highly induced miRNA in hypoxia [35]. The lack of impairment of miR-210 maturation even after the decrease of many miRNA biogenesis proteins in hypoxia could be due to robustly increased transcription of the miR-210 gene in hypoxia. Recently deep sequencing of miRNA populations in hypoxic and normoxic HUVECs also showed no significant difference in the majority of miRNA species [53]. Camps et al. (2014) reported down regulation of Dicer mRNA in hypoxia but failed to see a global down regulation of miRNAs in hypoxia compared to normoxia using small RNA sequencing techniques [48]. They reported that 41 miRNAs were up regulated and 28 miRNAs were down regulated in hypoxia compared to normoxia in MCF7 cells [48]. This work provides further support for Dicer down regulation by hypoxia but minimal influences on miRNA maturation.

In MCF7 cells only a modest decrease in mature and precursor levels of let-7a and miR-21 was seen after exposure to hypoxia, and we did not see an accumulation of pre-let-7a or pre-miR-21 in hypoxia. A significant reduction (P = 0.03) in mature miR-185 was observed in hypoxia in MCF7 cells, but there was no accumulation of precursors. These miRNAs (let-7a, miR-21 and miR-185) were chosen as previous reports showed they were Dicer dependent miRNAs and mature levels decreased with Dicer inhibition [44-46].

The use of knockout murine models has provided compelling evidence for the essential role of Dicer in mature miRNA production. However, it has proven more difficult to clearly see such critical influences in a variety of experiments that have utilised siRNA mediated Dicer suppression [44,54]. Others have previously explored the surprising stability of some miRNAs with an average miRNA half-life of 119 h [55], and this may largely explain the lack of changes in mature miRNA levels after 48 h of hypoxia or Dicer suppression by siRNA. The operation of Dicer independent pathways in the generation of miRNAs has been well described for a small number of specific miRNAs [56-58] but seems a less plausible explanation for the lack of influence of Dicer manipulations on global alterations in miRNA abundance. Another possibility is that the levels of proteins such as Dicer are not rate limiting for the production of miRNAs under these conditions. These observations suggest that early and immediate responses to hypoxic stresses are not likely to be mediated by global alterations in mature miRNA levels.

During the course of this work, Ho et al. (2012) also described a reduction in Dicer levels after exposure to hypoxia, and reported a decrease in the levels of several mature miRNAs and an accumulation of their precursor-miRNAs in HUVEC cells [45]. Whilst we were unable to replicate these findings using precisely controlled levels of hypoxia, this may suggest that in particular cells or conditions of hypoxic exposure, the hypoxic repression of Dicer can have important influences on miRNA biogenesis.

Whilst hypoxia exerted only modest effects on the production of mature miRNAs, we did see a significant influence of hypoxia on the operation of an exogenously introduced precursor miRNA. The processing and function of miR-200b was reduced in hypoxia when compared to normoxia. Dicer and TARBP2 proteins are involved in the processing of a precursor miRNA in to a mature miRNA and the reduction of both these proteins in hypoxia would likely have impacted on the maturation of the pre-miR-200b.

This work points towards the operation of non-HIF but HIF hydroxylase dependent pathways by which hypoxia controls reductions in gene expression. This effect of hypoxia appears to operate via several mechanisms of regulation and we have shown important influences of the HIF hydroxylase PHD2 and of the hypoxically regulated miRNAs miR-103 and miR-107 in this regulation. Furthermore, there appears to be co-ordinated regulation of several of the proteins involved in miRNA biogenesis under hypoxic and other conditions. However, influences on mature miRNA production were more modest than might have been anticipated from the substantial hypoxic reductions in protein expression of Dicer, Drosha, TARBP2 and XPO5. The breadth of this effect suggests a further and important interface between oxygen availability and gene expression.

## Conclusion

These observations show an important interface between oxygen availability and gene expression via alterations in the levels of proteins critical for miRNA generation. This may provide a mechanistic explanation for the reduced levels of miRNAs observed in some cancers and provides evidence for the existence of a co-ordinated mechanism of repression of levels of miRNA biogenesis proteins in response to hypoxia. There is a PHD2 dependent, but HIF independent, mechanism of repression of these proteins under hypoxic conditions. These results also provide further support for the existence of feedback mechanisms in the regulation of the miRNA biogenesis pathway. Despite such mechanisms, short term modulation of miRNA biogenesis in breast cancer cells only leads to modest effects on miRNA levels in keeping with their significant stability.

## Abbreviations

HIF: hypoxia inducible factor; TARPB2: TAR RNA-binding protein 2; DGCR8: DiGeorge syndrome critical region gene 8; XPO5: Exportin 5; RT PCR: reverse transcription polymerase chain reaction; PHD2: prolyl hydroxylase domain 2; miRNA: microRNA; DDX5: DEAD/H box 5; RISC: RNA induced silencing complex; DMOG: dimethyloxalylglycine; DFO: desferrioxamine; siRNA: small inhibitory ribonucleic acid; ZEB1: Zinc finger E box-binding homeobox 1; RL: Renilla luciferase; cDNA: complementary DNA; HUVEC: human umbilical vein endothelial cells; FIH-1: factor inhibiting HIF-1; SIP1: SMN interacting protein 1; AGO2: argonaute 2.

## Competing interests

The authors declare that they have no competing interests.

## Authors’ contributions

VB undertook all of the experimental work, prepared the results figures and analysis and drafted the manuscript. MM supervised the experimental work, participated in the design and interpretation of experiments and drafted the manuscript. JG conceived the study, its design and coordination, supervised the experimental work and drafted the manuscript. All authors read and approved the final manuscript.

## Pre-publication history

The pre-publication history for this paper can be accessed here:

http://www.biomedcentral.com/1471-2407/14/533/prepub

## Supplementary Material

Additional file 1: Table S1 List of siRNA sequences. **Table S2.** miRNAs significantly up regulated in MCF7 cells after exposure to hypoxia (0.1% O_2_ for 48 h). **Table S3.** miRNAs significantly down regulated in MCF7 cells after exposure to hypoxia (0.1% O_2_ for 48 h). **Figure S1.** Scatter plot of probe intensities of miRNAs expressed in MCF7 cells after hypoxia (0.1% O_2_ 48 h) vs. normoxia. ▼indicates miRNAs that were significantly down regulated in hypoxia, ▲indicates miRNAs that were significantly up regulated in hypoxia. **Figure S2.** Scatter plot of ratios between pre-miRNA/mature miRNA expression in MCF7 cells after hypoxia (0.1% O_2_ 48 h) vs. normoxia. **Figure S3.** Scatter plot of ratios between pre-miRNA/mature miRNA in MCF7 cells after Dicer inhibition by transient transfection with siRNAs vs. normoxia. **Figure S4.** Dicer protein expression in MCF7 cells after Dicer inhibition by siRNA compared with control siRNA. **Figure S5.** Mature and precursor miR-185 and miR-21 expression in HUVECs in hypoxia (1% O_2_ for 24 h) vs. normoxia. **Figure S6.** Mature and precursor miR-185 and miR-21 expression in HUVECs in hypoxia (1% O_2_ for 48 h) vs. normoxia. Click here for file

## References

[B1] Reynolds TY Rockwell S Glazer PM Genetic instability induced by the tumor microenvironment Cancer Res 1996 56 24 5754 5757 8971187

[B2] Thomlinson RH Gray LH The histological structure of some human lung cancers and the possible implications for radiotherapy Br J Cancer 1955 9 4 539 549 10.1038/bjc.1955.55 13304213 PMC2073776

[B3] Vaupel P Kallinowski F Okunieff P Blood flow, oxygen and nutrient supply, and metabolic microenvironment of human tumors: a review Cancer Res 1989 49 23 6449 6465 2684393

[B4] Harris AL Hypoxia–a key regulatory factor in tumour growth Nat Rev Cancer 2002 2 1 38 47 10.1038/nrc704 11902584

[B5] Lee Y Kim M Han J Yeom KH Lee S Baek SH Kim VN MicroRNA genes are transcribed by RNA polymerase II EMBO J 2004 23 20 4051 4060 10.1038/sj.emboj.7600385 15372072 PMC524334

[B6] Borchert GM Lanier W Davidson BL RNA polymerase III transcribes human microRNAs Nat Struct Mol Biol 2006 13 12 1097 1101 10.1038/nsmb1167 17099701

[B7] Han J Lee Y Yeom KH Kim YK Jin H Kim VN The Drosha-DGCR8 complex in primary microRNA processing Genes Dev 2004 18 24 3016 3027 10.1101/gad.1262504 15574589 PMC535913

[B8] Kim VN MicroRNA precursors in motion: exportin-5 mediates their nuclear export Trends Cell Biol 2004 14 4 156 159 10.1016/j.tcb.2004.02.006 15134074

[B9] Grishok A Pasquinelli AE Conte D Li N Parrish S Ha I Baillie DL Fire A Ruvkun G Mello CC Genes and mechanisms related to RNA interference regulate expression of the small temporal RNAs that control C. elegans developmental timing Cell 2001 106 1 23 34 10.1016/S0092-8674(01)00431-7 11461699

[B10] Hutvagner G McLachlan J Pasquinelli AE Balint E Tuschl T Zamore PD A cellular function for the RNA-interference enzyme Dicer in the maturation of the let-7 small temporal RNA Science 2001 293 5531 834 838 10.1126/science.1062961 11452083

[B11] Chendrimada TP Gregory RI Kumaraswamy E Norman J Cooch N Nishikura K Shiekhattar R TRBP recruits the Dicer complex to Ago2 for microRNA processing and gene silencing Nature 2005 436 7051 740 744 10.1038/nature03868 15973356 PMC2944926

[B12] Haase AD Jaskiewicz L Zhang H Laine S Sack R Gatignol A Filipowicz W TRBP, a regulator of cellular PKR and HIV-1 virus expression, interacts with Dicer and functions in RNA silencing EMBO Rep 2005 6 10 961 967 10.1038/sj.embor.7400509 16142218 PMC1369185

[B13] Khvorova A Reynolds A Jayasena SD Functional siRNAs and miRNAs exhibit strand bias Cell 2003 115 2 209 216 10.1016/S0092-8674(03)00801-8 14567918

[B14] Lim LP Lau NC Weinstein EG Abdelhakim A Yekta S Rhoades MW Burge CB Bartel DP The microRNAs of Caenorhabditis elegans Genes Dev 2003 17 8 991 1008 10.1101/gad.1074403 12672692 PMC196042

[B15] Zhou H Huang X Cui H Luo X Tang Y Chen S Wu L Shen N miR-155 and its star-form partner miR-155* cooperatively regulate type I interferon production by human plasmacytoid dendritic cells Blood 2010 116 26 5885 5894 10.1182/blood-2010-04-280156 20852130

[B16] Bartel DP MicroRNAs: target recognition and regulatory functions Cell 2009 136 2 215 233 10.1016/j.cell.2009.01.002 19167326 PMC3794896

[B17] Brennecke J Hipfner DR Stark A Russell RB Cohen SM bantam encodes a developmentally regulated microRNA that controls cell proliferation and regulates the proapoptotic gene hid in Drosophila Cell 2003 113 1 25 36 10.1016/S0092-8674(03)00231-9 12679032

[B18] Xu P Vernooy SY Guo M Hay BA The Drosophila microRNA Mir-14 suppresses cell death and is required for normal fat metabolism Curr Biol 2003 13 9 790 795 10.1016/S0960-9822(03)00250-1 12725740

[B19] Calin GA Dumitru CD Shimizu M Bichi R Zupo S Noch E Aldler H Rattan S Keating M Rai K Rassenti L Kipps T Negrini M Bullrich F Croce CM Frequent deletions and down-regulation of micro- RNA genes miR15 and miR16 at 13q14 in chronic lymphocytic leukemia Proc Natl Acad Sci U S A 2002 99 24 15524 15529 10.1073/pnas.242606799 12434020 PMC137750

[B20] Calin GA Sevignani C Dumitru CD Hyslop T Noch E Yendamuri S Shimizu M Rattan S Bullrich F Negrini M Croce CM Human microRNA genes are frequently located at fragile sites and genomic regions involved in cancers Proc Natl Acad Sci U S A 2004 101 9 2999 3004 10.1073/pnas.0307323101 14973191 PMC365734

[B21] Deng S Calin GA Croce CM Coukos G Zhang L Mechanisms of microRNA deregulation in human cancer Cell Cycle 2008 7 17 2643 2646 10.4161/cc.7.17.6597 18719391

[B22] Greither T Grochola LF Udelnow A Lautenschlager C Wurl P Taubert H Elevated expression of microRNAs 155, 203, 210 and 222 in pancreatic tumors is associated with poorer survival Int J Cancer 2010 126 1 73 80 10.1002/ijc.24687 19551852

[B23] Iorio MV Ferracin M Liu CG Veronese A Spizzo R Sabbioni S Magri E Pedriali M Fabbri M Campiglio M Menard S Palazzo JP Rosenberg A Musiani P Volinia S Nenci I Calin GA Querzoli P Negrini M Croce CM MicroRNA gene expression deregulation in human breast cancer Cancer Res 2005 65 16 7065 7070 10.1158/0008-5472.CAN-05-1783 16103053

[B24] Lu J Getz G Miska EA Alvarez-Saavedra E Lamb J Peck D Sweet-Cordero A Ebert BL Mak RH Ferrando AA Downing JR Jacks T Horvitz HR Golub TR MicroRNA expression profiles classify human cancers Nature 2005 435 7043 834 838 10.1038/nature03702 15944708

[B25] Thomson JM Newman M Parker JS Morin-Kensicki EM Wright T Hammond SM Extensive post-transcriptional regulation of microRNAs and its implications for cancer Genes Dev 2006 20 16 2202 2207 10.1101/gad.1444406 16882971 PMC1553203

[B26] Siomi H Siomi MC Posttranscriptional regulation of microRNA biogenesis in animals Mol Cell 2010 38 3 323 332 10.1016/j.molcel.2010.03.013 20471939

[B27] Horikawa Y Wood CG Yang H Zhao H Ye Y Gu J Lin J Habuchi T Wu X Single nucleotide polymorphisms of microRNA machinery genes modify the risk of renal cell carcinoma Clin Cancer Res 2008 14 23 7956 7962 10.1158/1078-0432.CCR-08-1199 19047128 PMC2650498

[B28] Merritt WM Lin YG Han LY Kamat AA Spannuth WA Schmandt R Urbauer D Pennacchio LA Cheng JF Nick AM Deavers MT Mourad-Zeidan A Wang H Mueller P Lenburg ME Gray JW Mok S Birrer MJ Lopez-Berestein G Coleman RL Bar-Eli M Sood AK Dicer, Drosha, and outcomes in patients with ovarian cancer N Engl J Med 2008 359 25 2641 2650 10.1056/NEJMoa0803785 19092150 PMC2710981

[B29] Karube Y Tanaka H Osada H Tomida S Tatematsu Y Yanagisawa K Yatabe Y Takamizawa J Miyoshi S Mitsudomi T Takahashi T Reduced expression of Dicer associated with poor prognosis in lung cancer patients Cancer Sci 2005 96 2 111 115 10.1111/j.1349-7006.2005.00015.x 15723655 PMC11158408

[B30] Kumar MS Pester RE Chen CY Lane K Chin C Lu J Kirsch DG Golub TR Jacks T Dicer1 functions as a haploinsufficient tumor suppressor Genes Dev 2009 23 23 2700 2704 10.1101/gad.1848209 19903759 PMC2788328

[B31] Kuehbacher A Urbich C Dimmeler S Targeting microRNA expression to regulate angiogenesis Trends Pharmacol Sci 2008 29 1 12 15 10.1016/j.tips.2007.10.014 18068232

[B32] Kulshreshtha R Ferracin M Wojcik SE Garzon R Alder H Agosto-Perez FJ Davuluri R Liu CG Croce CM Negrini M Calin GA Ivan M A microRNA signature of hypoxia Mol Cell Biol 2007 27 5 1859 1867 10.1128/MCB.01395-06 17194750 PMC1820461

[B33] Marsit CJ Eddy K Kelsey KT MicroRNA responses to cellular stress Cancer Res 2006 66 22 10843 10848 10.1158/0008-5472.CAN-06-1894 17108120

[B34] Caruso P MacLean MR Khanin R McClure J Soon E Southgate M MacDonald RA Greig JA Robertson KE Masson R Denby L Dempsie Y Long L Morrell NW Baker AH Dynamic changes in lung microRNA profiles during the development of pulmonary hypertension due to chronic hypoxia and monocrotaline Arterioscler Thromb Vasc Biol 2010 30 4 716 723 10.1161/ATVBAHA.109.202028 20110569

[B35] Camps C Buffa FM Colella S Moore J Sotiriou C Sheldon H Harris AL Gleadle JM Ragoussis J hsa-miR-210 Is induced by hypoxia and is an independent prognostic factor in breast cancer Clin Cancer Res 2008 14 5 1340 1348 10.1158/1078-0432.CCR-07-1755 18316553

[B36] Elvidge GP Glenny L Appelhoff RJ Ratcliffe PJ Ragoussis J Gleadle JM Concordant regulation of gene expression by hypoxia and 2-oxoglutarate-dependent dioxygenase inhibition: the role of HIF-1alpha, HIF-2alpha, and other pathways J Biol Chem 2006 281 22 15215 15226 10.1074/jbc.M511408200 16565084

[B37] Gregory PA Bert AG Paterson EL Barry SC Tsykin A Farshid G Vadas MA Khew-Goodall Y Goodall GJ The miR-200 family and miR-205 regulate epithelial to mesenchymal transition by targeting ZEB1 and SIP1 Nat Cell Biol 2008 10 5 593 601 10.1038/ncb1722 18376396

[B38] Edgar R Domrachev M Lash AE Gene Expression Omnibus: NCBI gene expression and hybridization array data repository Nucleic Acids Res 2002 30 1 207 210 10.1093/nar/30.1.207 11752295 PMC99122

[B39] Woo KJ Lee TJ Park JW Kwon TK Desferrioxamine, an iron chelator, enhances HIF-1alpha accumulation via cyclooxygenase-2 signaling pathway Biochem Biophys Res Commun 2006 343 1 8 14 10.1016/j.bbrc.2006.02.116 16527254

[B40] Appelhoff RJ Tian YM Raval RR Turley H Harris AL Pugh CW Ratcliffe PJ Gleadle JM Differential function of the prolyl hydroxylases PHD1, PHD2, and PHD3 in the regulation of hypoxia-inducible factor J Biol Chem 2004 279 37 38458 38465 10.1074/jbc.M406026200 15247232

[B41] Martello G Rosato A Ferrari F Manfrin A Cordenonsi M Dupont S Enzo E Guzzardo V Rondina M Spruce T Parenti AR Daidone MG Bicciato S Piccolo S A MicroRNA targeting dicer for metastasis control Cell 2010 141 7 1195 1207 10.1016/j.cell.2010.05.017 20603000

[B42] Forman JJ Legesse-Miller A Coller HA A search for conserved sequences in coding regions reveals that the let-7 microRNA targets Dicer within its coding sequence Proc Natl Acad Sci U S A 2008 105 39 14879 14884 10.1073/pnas.0803230105 18812516 PMC2567461

[B43] Melo SA Ropero S Moutinho C Aaltonen LA Yamamoto H Calin GA Rossi S Fernandez AF Carneiro F Oliveira C Ferreira B Liu CG Villanueva A Capella G Schwartz S Jr Shiekhattar R Esteller M A TARBP2 mutation in human cancer impairs microRNA processing and DICER1 function Nat Genet 2009 41 3 365 370 10.1038/ng.317 19219043 PMC4509508

[B44] Suarez Y Fernandez-Hernando C Pober JS Sessa WC Dicer dependent microRNAs regulate gene expression and functions in human endothelial cells Circ Res 2007 100 8 1164 1173 10.1161/01.RES.0000265065.26744.17 17379831

[B45] Ho JJ Metcalf JL Yan MS Turgeon PJ Wang JJ Chalsev M Petruzziello-Pellegrini TN Tsui AK He JZ Dhamko H Man HS Robb GB Teh BT Ohh M Marsden PA Functional importance of Dicer protein in the adaptive cellular response to hypoxia J Biol Chem 2012 287 34 29003 29020 10.1074/jbc.M112.373365 22745131 PMC3436557

[B46] Bu Y Lu C Bian C Wang J Li J Zhang B Li Z Brewer G Zhao RC Knockdown of Dicer in MCF-7 human breast carcinoma cells results in G1 arrest and increased sensitivity to cisplatin Oncol Rep 2009 21 1 13 17 19082437

[B47] Wu C So J Davis-Dusenbery BN Qi HH Bloch DB Shi Y Lagna G Hata A Hypoxia potentiates microRNA-mediated gene silencing through posttranslational modification of Argonaute2 Mol Cell Biol 2011 31 23 4760 4774 10.1128/MCB.05776-11 21969601 PMC3232924

[B48] Camps C Saini HK Mole DR Choudhry H Reczko M Guerra-Assuncao JA Tian YM Buffa FM Harris AL Hatzigeorgiou AG Enright AJ Ragoussis J Integrated analysis of microRNA and mRNA expression and association with HIF binding reveals the complexity of microRNA expression regulation under hypoxia Mol Cancer 2014 13 28 10.1186/1476-4598-13-28 24517586 PMC3928101

[B49] Romero-Ruiz A Bautista L Navarro V Heras-Garvin A March-Diaz R Castellano A Gomez-Diaz R Castro MJ Berra E Lopez-Barneo J Pascual A Prolyl hydroxylase-dependent modulation of eukaryotic elongation factor 2 activity and protein translation under acute hypoxia J Biol Chem 2012 287 12 9651 9658 10.1074/jbc.M111.299180 22308030 PMC3308822

[B50] Kulshreshtha R Ferracin M Negrini M Calin GA Davuluri RV Ivan M Regulation of microRNA expression: the hypoxic component Cell Cycle 2007 6 12 1426 1431 17582223

[B51] Han J Pedersen JS Kwon SC Belair CD Kim YK Yeom KH Yang WY Haussler D Blelloch R Kim VN Posttranscriptional crossregulation between Drosha and DGCR8 Cell 2009 136 1 75 84 10.1016/j.cell.2008.10.053 19135890 PMC2680683

[B52] Smibert P Yang JS Azzam G Liu JL Lai EC Homeostatic control of Argonaute stability by microRNA availability Nat Struct Mol Biol 2013 20 789 795 10.1038/nsmb.2606 23708604 PMC3702675

[B53] Voellenkle C Rooij J Guffanti A Brini E Fasanaro P Isaia E Croft L David M Capogrossi MC Moles A Felsani A Martelli F Deep-sequencing of endothelial cells exposed to hypoxia reveals the complexity of known and novel microRNAs RNA 2012 18 3 472 484 10.1261/rna.027615.111 22282338 PMC3285935

[B54] Yang JS Lai EC Alternative miRNA biogenesis pathways and the interpretation of core miRNA pathway mutants Mol Cell 2011 43 6 892 903 10.1016/j.molcel.2011.07.024 21925378 PMC3176435

[B55] Gantier MP McCoy CE Rusinova I Saulep D Wang D Xu D Irving AT Behlke MA Hertzog PJ Mackay F Williams BR Analysis of microRNA turnover in mammalian cells following Dicer1 ablation Nucleic Acids Res 2011 39 13 5692 5703 10.1093/nar/gkr148 21447562 PMC3141258

[B56] Langenberger D Cakir MV Hoffmann S Stadler PF Dicer-processed small RNAs: rules and exceptions J Exp Zool B Mol Dev Evol 2012 320 1 35 46 23165937 10.1002/jez.b.22481

[B57] Cifuentes D Xue H Taylor DW Patnode H Mishima Y Cheloufi S Ma E Mane S Hannon GJ Lawson ND Wolfe SA Giraldez AJ A novel miRNA processing pathway independent of Dicer requires Argonaute2 catalytic activity Science 2010 328 5986 1694 1698 10.1126/science.1190809 20448148 PMC3093307

[B58] Cheloufi S Dos Santos CO Chong MM Hannon GJ A dicer-independent miRNA biogenesis pathway that requires Ago catalysis Nature 2010 465 7298 584 589 10.1038/nature09092 20424607 PMC2995450

