# Supplementary material for: Hypoxia represses microRNA biogenesis proteins in breast cancer cells
Source: BMC Cancer. 2014 Jul 22;14:533. doi: 10.1186/1471-2407-14-533 (PMC4223767; doi:10.1186/1471-2407-14-533)
Supplement: Additional file 1: Table S1 — List of siRNA sequences. Table S2. miRNAs significantly up regulated in MCF7 cells after exposure to hypoxia (0.1% O2 for 48 h). Table S3. miRNAs significantly down regulated in MCF7 cells after exposure to hypoxia (0.1% O2 for 48 h). Figure S1. Scatter plot of probe intensities of miRNAs expressed in MCF7 cells after hypoxia (0.1% O2 48 h) vs. normoxia. ▼indicates miRNAs that were significantly down regulated in hypoxia, ▲indicates miRNAs that were significantly up regulated in hypoxia. Figure S2. Scatter plot of ratios between pre-miRNA/mature miRNA expression in MCF7 cells after hypoxia (0.1% O2 48 h) vs. normoxia. Figure S3. Scatter plot of ratios between pre-miRNA/mature miRNA in MCF7 cells after Dicer inhibition by transient transfection with siRNAs vs. normoxia. Figure S4. Dicer protein expression in MCF7 cells after Dicer inhibition by siRNA compared with control siRNA. Figure S5. Mature and precursor miR-185 and miR-21 expression in HUVECs in hypoxia (1% O2 for 24 h) vs. normoxia. Figure S6. Mature and precursor miR-185 and miR-21 expression in HUVECs in hypoxia (1% O2 for 48 h) vs. normoxia. [file 1471-2407-14-533-S1.pdf]

Additional file 1.pdf

Supplementary tables and figures

Table S1 List of siRNA sequences.

Table S2 miRNAs significantly up regulated in MCF7 cells after exposure to hypoxia (0.1% O_2 for 48 h). Differentially expressed genes were identified using ANOVA ($p<0.05$).

Table S3 miRNAs significantly down regulated in MCF7 cells after exposure to hypoxia (0.1% O_2 for 48 h). Differentially expressed genes were identified using ANOVA ($p<0.05$).

Figure S1 Scatter plot of probe intensities of miRNAs expressed in MCF7 cells after hypoxia (0.1% O_2 48 h) vs. normoxia. ▼ indicates miRNAs that were significantly down regulated in hypoxia, ▲ indicates miRNAs that were significantly up regulated in hypoxia. Differentially expressed genes were identified using ANOVA and FDR ($p<0.05$).

Figure S2 Scatter plot of ratios between pre-miRNA/mature miRNA expression in MCF7 cells after hypoxia (0.1% O_2 48 h) vs. normoxia.

Figure S3 Scatter plot of ratios between pre-miRNA/mature miRNA in MCF7 cells after Dicer inhibition by transient transfection with siRNAs vs. normoxia.

Figure S4 Dicer protein expression in MCF7 cells after Dicer inhibition by siRNA compared with control siRNA.

Figure S5 Mature and precursor miR-185 and miR-21 expression in hypoxia (1% O_2 for 24 h) vs. normoxia A**,** Pre-miR-185 and miR-185 expression, precursor/mature ratio in HUVECs exposed to hypoxia (1% O_2 for 24 h) vs. normoxia. B, Pre-miR-21 and miR-21 expression, precursor/mature ratio in HIVECs exposed to hypoxia (1% O_2 for 24 h) vs. normoxia**.** Data represent normalized mean ±S.E (error bars) (n=3). miRNA levels were analysed by RT-PCR and normalised to RNU6B levels.

Figure S6 Mature and precursor miR-185 and miR-21 expression in hypoxia (1% O_2 for 48 h) vs. normoxia A**,** Pre-miR-185 and miR-185 expression, precursor/mature ratio in HUVECs exposed to hypoxia (1% O_2 for 48 h) vs. normoxia. B, Pre-miR-21 and miR-21 expression, precursor/mature ratio in HIVECs exposed to hypoxia (1% O_2 for 48 h) vs. normoxia**.** Data represent normalized mean ±S.E (error bars) (n=3). miRNA levels were analysed by RT-PCR and normalised to RNU6B levels.

Table S1 List of siRNA sequences.

siRNA	siRNA sequence	Company	Reference
DICER	5’-UCCAGAGCUGCUUCAAGCATT-3’<br><br>5’-UGCUUGAAGCAGCUCUGGATT-3’	Shanghai GenePharma Co., Ltd	(Chu et al., 2010)
DROSHA	5-ACGAAGCUCGAUGAAGAUUUATT-3’<br><br>5’-UAAAUCUUCAUCGAGCUUCGUTT-3’	Shanghai GenePharma Co., Ltd	(Han et al., 2012)
TARBP2	5’-AAUUCACCAUGACCUGUCGAGTT-3’<br><br>5’-CUCGACAGGUCAUGGUGAAUUTT-3’	Shanghai GenePharma Co., Ltd	(Christensen et al., 2007)
HIF-1α	5’-CUGAUGACCAGCAACUUGAdTdT-3’<br><br>5’-UCAAGUUGCUGGUCAUCAGdTdT-3’	Dharmacon, Lafayette, CO, USA	(Sowter et al., 2003)
HIF-2α	5’-CAGCAUCUUUGAUAGCAGUdTdT-3’<br><br>5’-ACUGCUAUCAAAGAUGCUGdTdT-3’	Dharmacon, Lafayette, CO, USA	(Sowter et al., 2003)
PHD1	5’-CUAUAUCGUGCCCUGAUGTT-3’<br><br>5’-CAUGCAGGGCACGAUAUAGUC-3’	Shanghai GenePharma Co., Ltd	(Appelhoff et al., 2004)
PHD2	5’-GGACAUCCGAGGCGAUAAGTT-3’<br><br>5’-CUUAUCGCCUCGGAUGUCCTT-3’	Shanghai GenePharma Co., Ltd	(Appelhoff et al., 2004)
FIH-1	5’-CAGCGGCGGAGGCUGUGGCTT-3’<br><br>5’-GCCACAGCCUCCGCCGCUGTT-3’	Shanghai GenePharma Co., Ltd	(Stolze et al., 2004)
Control siRNA	5’-UUCUCCGAACGUGUCACGUTT-3’<br><br>5’-ACGUGACACGUUCGGAGAATT-3’	Shanghai GenePharma Co., Ltd	

Table S2 miRNAs significantly up regulated in MCF7 cells after exposure to hypoxia (0.1% O_2 for 48 h). Listed were differentially expressed miRNAs with $p<0.05$ so determined by ANOVA.

**miRNA**	**Fold-Change**	**p-value**
hsa-miR-3193	24.85	0.0349
hsa-miR-210	8.05	0.0007
hsa-miR-181a-3p	7.33	0.0418
hsa-miR-27a-5p	5.93	0.0104
hsa-miR-23a-5p	3.01	0.0413
hsa-miR-193b-5p	2.76	0.0104
hsa-miR-1972	1.71	0.0309
hsa-miR-193b	1.68	0.0104

Table S3 miRNAs significantly down regulated in MCF7 cells after exposure to hypoxia (0.1% O_2 for 48 h). Listed were differentially expressed miRNAs with $p<0.05$ so determined by ANOVA.

**miRNA**	**Fold-Change**	**p-value**
hsa-miR-149	-2.19	0.0077
hsa-miR-205	-2.47	0.0307
hsa-miR-33b-3p	-5.12	0.0360
hsa-miR-4521	-7.35	0.0008

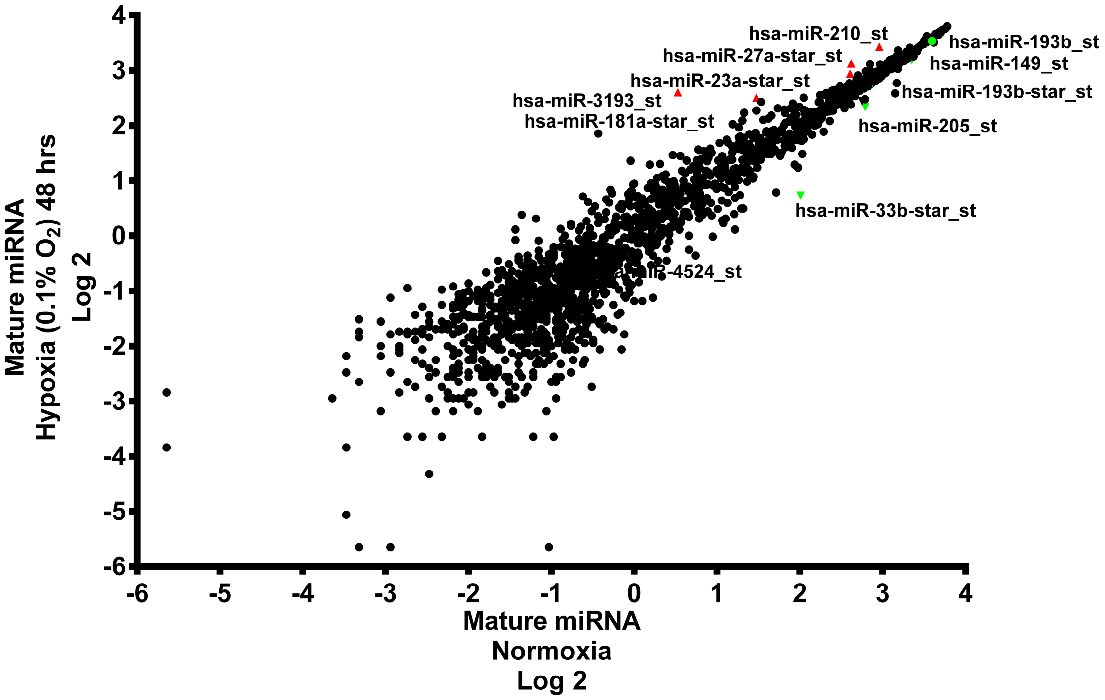

Figure S1 Microarray analysis. Scatter plot of probe intensities of miRNAs expressed in MCF7 cells after hypoxia (0.1% O_2 48 h) vs. normoxia. ▼ indicates miRNAs that were significantly down regulated in hypoxia, ▲ indicates miRNAs that were significantly up regulated in hypoxia. Differentially expressed genes were identified using ANOVA and FDR ($p<0.05$).

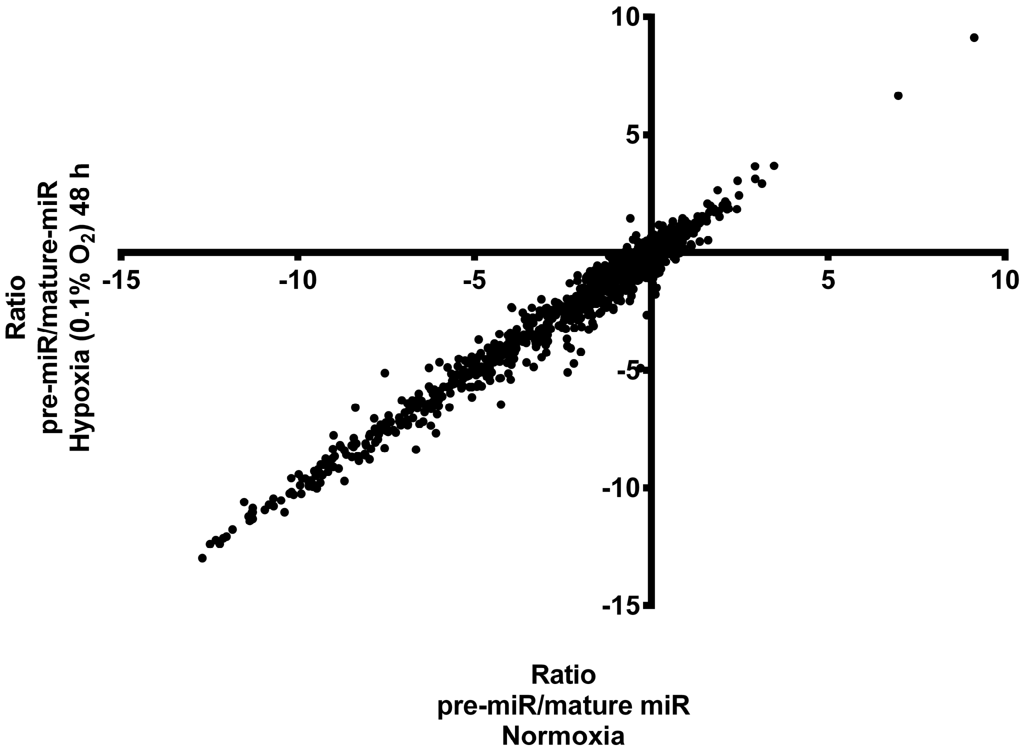

Figure S2 Scatter plot of ratios between pre-miRNA/mature miRNA levels in MCF7 cells after hypoxia (0.1% O_2 48 h) vs. normoxia as determined using microarray analysis.

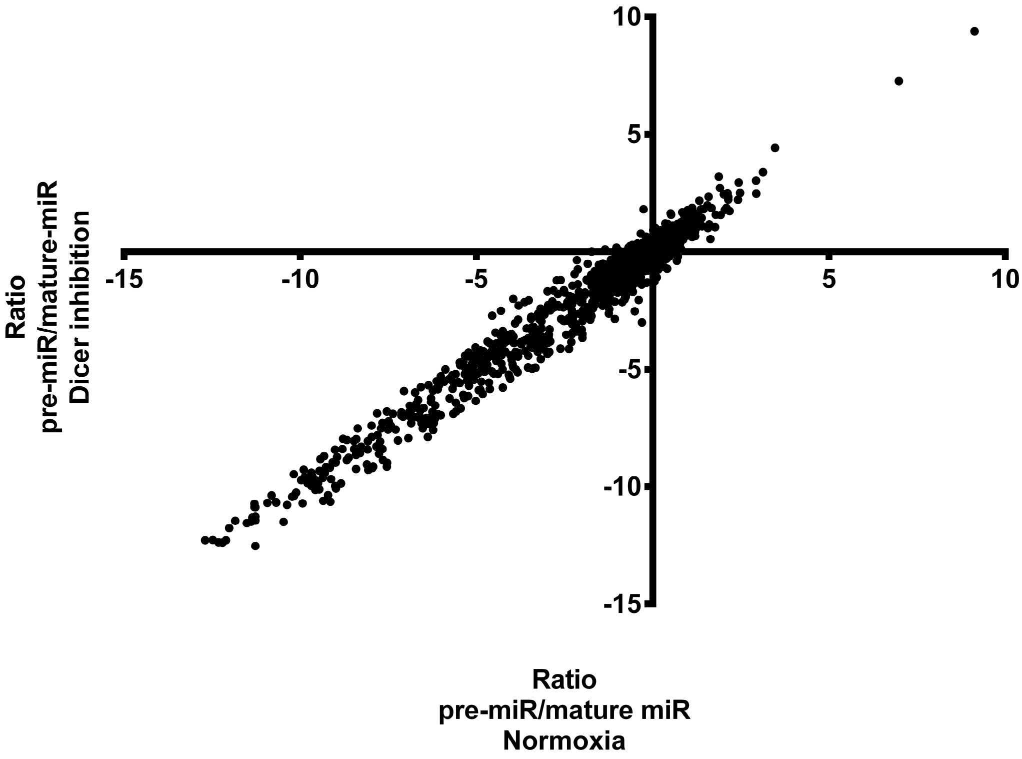

Figure S3 Scatter plot of ratios between pre-miRNA/mature miRNA in MCF7 cells after Dicer inhibition by transient transfection with siRNAs vs. normoxia.

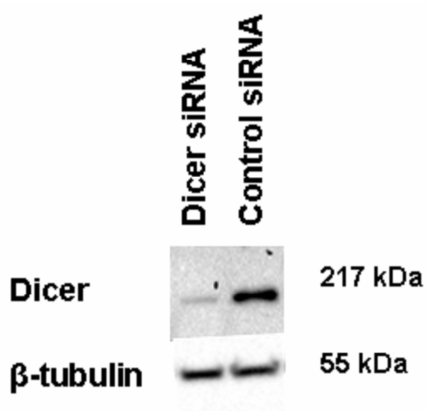

Figure S4 A, Dicer protein expression in MCF7 cells after Dicer inhibition by siRNA compared with control siRNA. β-tubulin was used as the loading control.

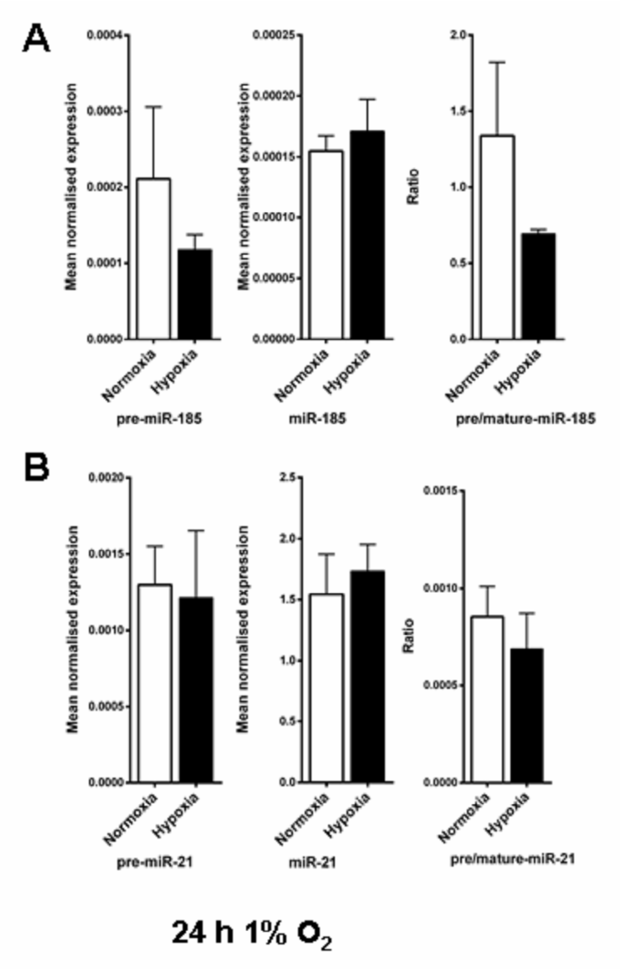

Figure S5 Mature and precursor miR-185 and miR-21 expression in hypoxia (1% O_2 for 24 h) vs. normoxia A**,** Pre-miR-185 and miR-185 expression, precursor/mature ratio in HUVECs exposed to hypoxia (1% O_2 for 24 h) vs. normoxia. B, Pre-miR-21 and miR-21 expression, precursor/mature ratio in HIVECs exposed to hypoxia (1% O_2 for 24 h) vs. normoxia**.** Data represent normalized mean ±S.E (error bars) (n=3). miRNA levels were analysed by RT-PCR and normalised to RNU6B levels.

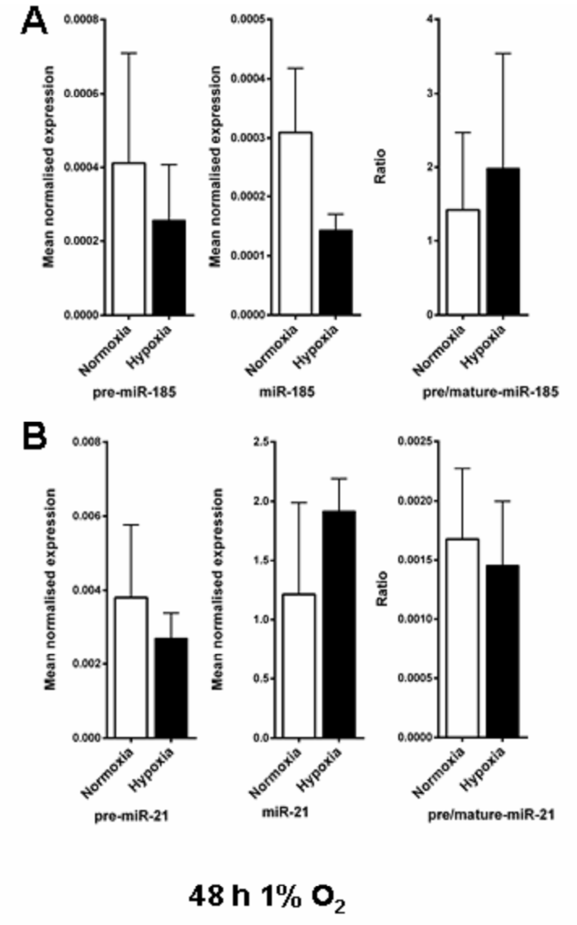

Figure S6 Mature and precursor miR-185 and miR-21 expression in hypoxia (1% O_2 for 48 h) vs. normoxia A**,** Pre-miR-185 and miR-185 expression, precursor/mature ratio in HUVECs exposed to hypoxia (1% O_2 for 48 h) vs. normoxia. B, Pre-miR-21 and miR-21 expression, precursor/mature ratio in HIVECs exposed to hypoxia (1% O_2 for 48 h) vs. normoxia**.** Data represent normalized mean ±S.E (error bars) (n=3). miRNA levels were analysed by RT-PCR and normalised to RNU6B levels.
